# Conflict Bear Translocation: Investigating Population Genetics and Fate of Bear Translocation in Dachigam National Park, Jammu and Kashmir, India

**DOI:** 10.1371/journal.pone.0132005

**Published:** 2015-08-12

**Authors:** Mukesh, Lalit Kumar Sharma, Samina Amin Charoo, Sambandam Sathyakumar

**Affiliations:** 1 Wildlife Institute of India, Chandrabani, Dehradun, 248 001, Uttarakhand, India; 2 Directorate of Extension, Indian Council of Forestry Research and Education, New Forest, Dehradun, -248006, Uttarakhand, India; 3 Department of Wildlife Protection, Forest Complex, Srinagar, 190 008, Jammu and Kashmir, India; Sichuan University, CHINA

## Abstract

The Asiatic black bear population in Dachigam landscape, Jammu and Kashmir is well recognized as one of the highest density bear populations in India. Increasing incidences of bear-human interactions and the resultant retaliatory killings by locals have become a serious threat to the survivorship of black bears in the Dachigam landscape. The Department of Wildlife Protection in Jammu and Kashmir has been translocating bears involved in conflicts, *henceforth ‘*conflict bears’ from different sites in Dachigam landscape to Dachigam National Park as a flagship activity to mitigate conflicts. We undertook this study to investigate the population genetics and the fate of bear translocation in Dachigam National Park. We identified 109 unique genotypes in an area of *ca*. 650 km^2^ and observed bear population under panmixia that showed sound genetic variability. Molecular tracking of translocated bears revealed that mostly bears (7 out of 11 bears) returned to their capture sites, possibly due to homing instincts or habituation to the high quality food available in agricultural croplands and orchards, while only four bears remained in Dachigam National Park after translocation. Results indicated that translocation success was most likely to be season dependent as bears translocated during spring and late autumn returned to their capture sites, perhaps due to the scarcity of food inside Dachigam National Park while bears translocated in summer remained in Dachigam National Park due to availability of surplus food resources. Thus, the current management practices of translocating conflict bears, without taking into account spatio-temporal variability of food resources in Dachigam landscape seemed to be ineffective in mitigating conflicts on a long-term basis. However, the study highlighted the importance of molecular tracking of bears to understand their movement patterns and socio-biology in tough terrains like Dachigam landscape.

## Introduction

In India, the Asiatic black bear (*Ursus thibetanus*) is distributed in the forested habitats of the Himalayas and hills of northeastern States covering an area of about 270,000 km^2^ with an estimated population of 5,400 to 6,700 individuals [1, 2, 3]. The ability of black bear to survive and negotiate man-made habitats such as plantations, orchards, cultivated areas, scrublands, and even villages to move between forested areas has not only resulted in its continuous distribution but also led to increasing bear-human conflicts [2].The Dachigam landscape (DL) falls in the central wildlife division of Kashmir region in the State of Jammu and Kashmir (J&K), India possess a mosaic of Protected Areas such as Dachigam National Park (DNP), City Forest National Park, Over-Aru Wildlife Sanctuary (WS), Thajwas WS and 10 Conservation Reserves *viz*., Brian-Nishat, Dara, Khonmoh, Khrew, Khiram, Shikargah, Wangat, Khangund, Panyer, Hajin along with croplands and human habitations placed in between forest patches [3]. The bear population in this landscape is recognized as one of the highest density populations of the Asiatic black bear in India [1]. However, this population is threatened due to retaliatory killings as a consequence of increasing incidences of bear-human conflicts [4] and also due to conversion of forested habitats to other land use types [5]. In DL, the current practice of managing bear-human conflicts involves capture of conflict bears from any part of DL and their translocation to DNP.

Fischer and Lindenmayer [6] compiled 180 studies on wildlife translocations and reported that only 5% of translocations were carried out to resolve human wildlife conflicts while studies documented that mere transloation of conflict bears from one place to other was not effective in reducing conflicts on long term basis without aversive conditioning [7, 8]. Furthermore, the patchy distribution of food resources can stimulate long range movements and extend bear home ranges [9]. Conversely, bears are reported to unoccasionally visit the same conflict sites in corn crops after translocation [10]. While Yosemite black bears were often reported to return to developed areas after being transported to undeveloped areas [11]. Several studies have reported the tendency of the translocated animals to return homeward from large distances *e*.*g*. White-tailed deer (*Odocoileus virginianus*) from 560 km distance [12], polar bear (*Ursus maritimus*) from 480 km distance [13], black bear (*Ursus americanus*) from 229 km distance [8, 14, 15], Asian elephant (*Elephas maximus*) from 100 km distance [16].

Due to complex biological and behavioral proclivities, managing human bear conflicts with simultaneously supporting bear’s populations is a daunting task. Studying bear movement patterns using traditional field techniques is difficult due to their crepuscular/nocturnal behavior, large home range sizes and their use of steep and rugged terrain. Therefore, molecular markers and non-invasive genetic sampling have been used in several studies to study the various important aspects of ecology and genetics of free ranging animals including gene flow [17], population genetic structure [18,19], population demography [20], genetic diversity [21,22], sex identification [23, 24], individual identification [18, 25] and evolutionary history [22, 26].

In India, translocations of conflict animals are increasingly employed to mitigate man-animal conflicts on a variety of large mammals. However, their overall effectiveness is conditional and still questionable. We undertook this study keeping three questions in mind *i*.*e*. 1). What is the fate of translocation after the conflict bears being caught elsewhere in the DL and released to DNP? 2). Do the translocated bears, if settle in DNP impact the population genetic structure of the resident population? 3). How translocated bears use Land use/land cover (LULC) classes in DL?

## Materials and Methods

### Ethical Statement

The majority of samples used in this study were hairs collected from hair snare stations placed in DNP and barbwire fencing deployed around the horticulture croplands. These samples were collected without animal handling and capture. Blood samples were collected from radio collared animals and from the bears translocated to DNP by the Department of Wildlife Protection, J&K to mitigate conflicts. Radio-collaring of black bears was conducted under the project ‘Ecology of the Asiatic black bear in Dachigam NP’ of Wildlife Institute of India. All necessary permissions for collection of biological samples of the Asiatic black bear were obtained from Principal Chief Wildlife Warden, J&K State, under the Wildlife (Protection) Act, 1972 of Government of India and J& K Wildlife (Protection) Act, 1978. The permissions define the conditions for collection of blood samples from the captured bears, which include an approved protocol and participation by the Park Officials and supervision by a qualified forest department Veterinarian. Capture operations were conducted by the trained team members including Park Officials, Veterinarians and wildlife biologists as per the protocols of the Wildlife Institute of India and Department of Wildlife Protection, J&K. This study was undertaken prior to the constitution of an Institute Animal Ethical Committee (IAEC) at the Wildlife Institute of India, Dehradun. However, all protocols of animal capture, handling and sample collection were as per the accordance and approved by Training Research Advisory Committee (TRAC) of Wildlife Institute of India.

### Study design, sample collection and DNA isolation

**Fig 1:**
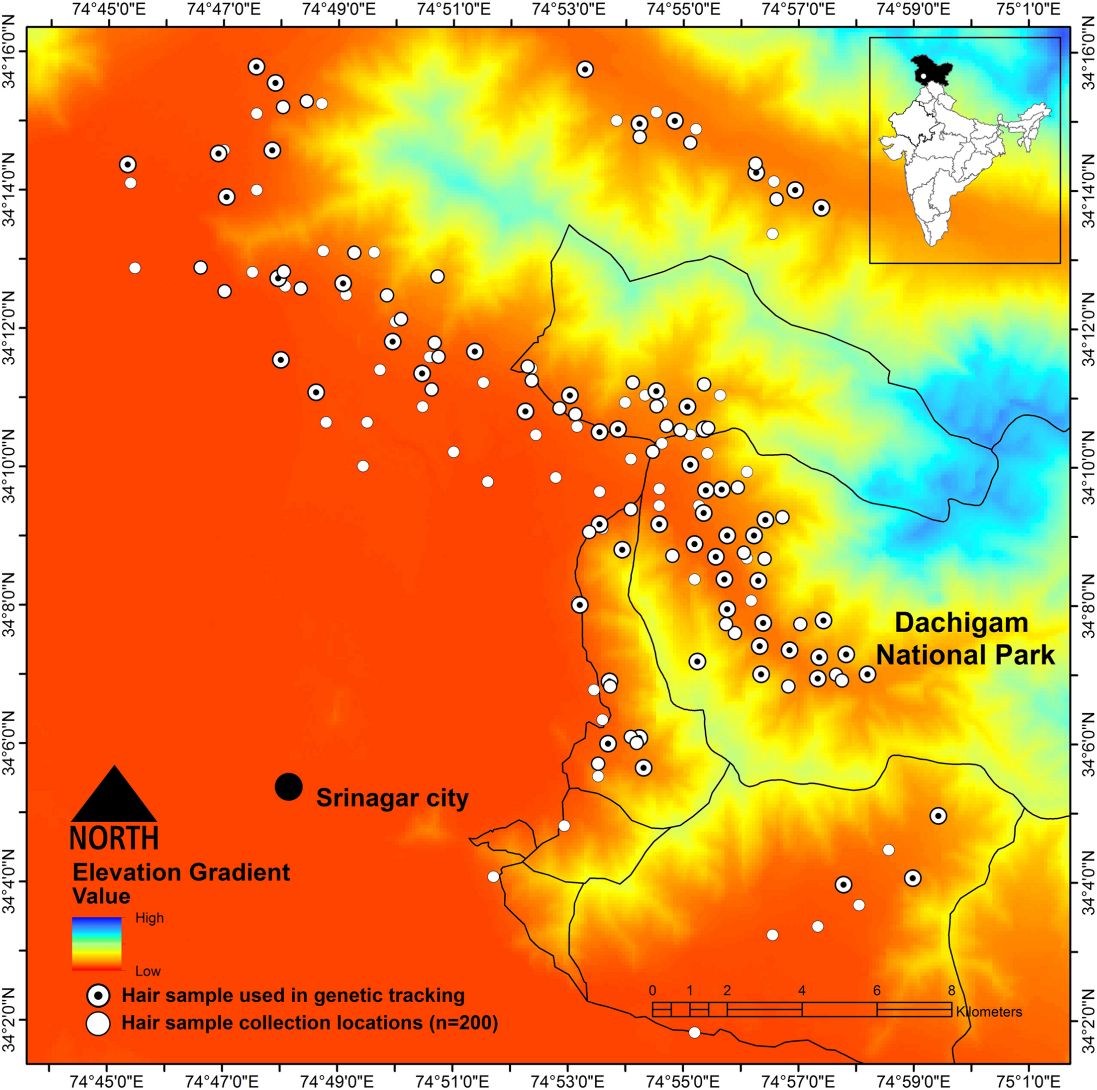
Digital elevation model of Dachigam National Park and other conservation reserves in Dachigam landscape, J& K. (all dots on map depicts the sampling location of individual hair tuft collected and black dots inside the sampling points represent position of translocated bears used in molecular tracking).

Intensive sampling was carried out in lower Dachigam during 2009 to 2011 where the protected area of *ca*.90 km^2^ was divided into 23 grids (2x2 km) for homogenous sampling [27, 28]. A hair snare station along with a camera trap station was placed in each grid and baited with bear attractants (honey/ putrid fruits) [27, 29]. In addition, hairs were also collected from the barbwire fencing of the horticulture croplands and camera traps were deployed to monitor the bear’s movements around the croplands. We collected blood samples from 18 bears, which include seven radio-collared bears and 11 conflict bears which were captured from different sites in DL by the Department of Wildlife Protection, J&K and translocated to DNP. A total of 200 individual hair tufts were collectively sampled from hair snare stations and from barbwire fencing of horticulture croplands in the surroundings of DNP (total sampling efforts spanning over an area of *ca*. 650 Km^2^, Fig 1). The genomic DNA was extracted from blood and hair samples using DNeasy Blood & Tissue Kit (Qiagen, Germany) following the manufacturer's instructions with minor modifications for hair samples [30].

### PCR, microsatellite genotyping and molecular sexing

We selected 20 microsatellites of which 10 loci (UT1, UT3, UT4, UT23, UT25, UT29, UT31, UT36, UT35 and UT38) were isolated from Formosan black bear (*Ursus thibetanus formosanus*) [31], eight (MSUT1, MSUT2, MSUT3, MSUT4, MSUT5, MSUT6, MSUT7, and MSUT8) from Japanese black bear (*Ursus thibetanus japonicas*) [32] and two (G10J and G10H) from American black bear (*Ursus americanus*) [21]. Independent PCR standardizations were attempted for amplification of each locus with blood DNA extracts and then primers were pooled for multiplexing with standardized conditions. Using Qiagen (Mainz, Germany) multiplex PCR kit, PCR reactions were set up in a 15 *μ*L of reaction volume containing 7.5 *μ*L of 2× Qiagen multiplex PCR master mix, 0.50 *μ*L of 10 *μ*M of each primer pair, 1 *μ*L of DNA elutant (∼ 20- 100ng) and remaining RNase-free water. The amplification conditions were 15 min initial heat activation of Hot Start (Mainz, Germany) *Taq* DNA polymerase at 95°C, followed by 35 cycles of denaturation at 94°C for 30 s, annealing at specific temperature (Table 1 for MP 1 to MP 6) for 90 s and extension at 72°C for 60 s with a final extension at 60°C for 30 min. Fluorescence-based genotyping was performed on ABI 3130 Genetic Analyzer (Applied Biosystem, Foster City, USA) and alleles were manually scored using GeneMapper software version 3.7 (Applied Biosystems, USA). Molecular sexing was attempted through simultaneous amplification of the partial fragments of *SRY* and *Amelogenin* genes in a single PCR and their profiles were viewed through capillary electrophoresis [23].

**Table 1:** Genetic polymorphism of 17 microsatellites screened with 18 reference blood DNA extracts of black bears. Annealing temperature (T_A_) for multiplexes–MP 1 (50°C), MP 2 (56°C), MP 3 (64°C), MP 4 (45°C), MP 5 (62°C) and MP 6 (55°C).

Locus	Reported size range	Success rate/MP	Na^1^	Ne^2^	Ho^3^	He^4^	PIC^5^	Fis^6^ (W&C)	P_ID_ ^7^ (locus)	P_ID_ sibs^8^ (locus)	P_ID_ ^9^ (Cum)	P_ID_ sib^10^ (Cum)	ADO^11^	FA^12^	F_Null_ ^13^
MSUT2 ^H^	77–91	100/MP 1	9	6.29	0.89	0.87	0.82	-0.03	4.4E-02	3.4E-01	4.4E-02	3.4E-01	0.00	0.00	-0.03
G10J ^H^	80–88	100/ MP 2	9	5.63	0.89	0.85	0.80	-0.05	5.4E-02	3.5E-01	2.4E-03	1.2E-01	0.00	0.00	-0.05
MSUT8 ^H^	106–110	94.44/ MP 1	8	5.40	0.94	0.84	0.79	-0.13	5.6E-02	3.6E-01	1.3E-04	4.3E-02	0.00	0.00	-0.09
MSUT4 ^H^	85–101	100/MP 4	7	4.47	0.89	0.80	0.74	-0.12	8.2E-02	3.8E-01	1.1E-05	1.6E-02	0.00	0.00	-0.08
UT1 ^H^ †	176–192	100/MP 3	6	3.38	0.44	0.72	0.66	0.39	1.3E-01	4.3E-01	1.4E-06	7.0E-03	0.00	0.00	0.20
UT4 ^H^ †	157–182	100/MP 2	5	3.00	0.83	0.69	0.61	-0.22	1.6E-01	4.6E-01	2.4E-07	3.2E-03	0.00	0.00	-0.12
UT35 ^H^ †	218–247	100/MP 5	4	2.95	0.94	0.68	0.60	-0.41	1.8E-01	4.6E-01	4.2E-08	1.5E-03	0.02	0.00	-0.19
MSUT7†	114–116	100/ MP 4	6	2.79	0.33	0.66	0.58	0.50	1.9E-01	4.8E-01	8.2E-09	7.2E-04	0.00	0.17	0.34
UT29	204–236	72.22/ MP 3	7	5.04	0.77	0.83	0.78	0.08	6.6E-02	3.7E-01	5.4E-10	2.6E-04	0.02	0.02	0.02
UT36	276–309	100/MP 5	3	1.58	0.33	0.38	0.34	0.12	4.3E-01	6.7E-01	2.3E-10	1.8E-04	0.00	0.00	0.12
MSUT6 †	183–193	94.44/ MP 1	8	4.59	0.29	0.81	0.75	0.64	7.6E-02	3.8E-01	1.8E-11	6.7E-05	0.00	0.00	0.46
UT25	314–333	100/MP 5	7	4.60	0.72	0.80	0.75	0.11	8.0E-02	3.8E-01	1.4E-12	2.5E-05	0.00	0.00	0.04
MSUT1†	170–174	94.44/ MP 6	5	1.55	0.18	0.37	0.34	0.53	4.3E-01	6.8E-01	6.1E-13	1.7E-05	0.00	0.00	0.42
UT38	196–232	94.44/MP 6	8	6.35	0.71	0.87	0.82	0.19	4.4E-02	3.4E-01	2.7E-14	5.8E-06	0.00	0.03	0.09
MSUT5 †	167–171	72.22/ MP 1	8	5.20	0.62	0.84	0.78	0.28	6.2E-02	3.6E-01	1.7E-15	2.1E-06	0.17	0.04	0.14
UT3	256–282	44.44/ MP 2	7	4.00	0.88	0.80	0.73	-0.10	8.8E-02	4.0E-01	1.5E-16	8.4E-07	0.00	0.00	ND
UT31	315–369	38.89/ MP 3	10	8.17	0.86	0.95	0.87	0.10	2.7E-02	3.2E-01	3.9E-18	2.7E-07	0.00	0.00	ND
Mean			6.88	4.41	0.68	0.75	0.69	0.11*							
SE			0.45	0.43	0.06	0.04	0.04	0.07							

1—observed number of alleles

2—effective number of alleles

3—observed heterozygosity

4—expected heterozygosity

5—polymorphic information content

6—inbreeding coefficient

7—probability of identity (locus)

8—probability of identity for sibs (locus)

9—probability of identity (cumulative)

10—probability of identity for sibs (cumulative)

11—allelic dropout rate

12—false allele rate

13—predicted frequencies of null alleles.

H—locus used for individual identification

† HWE deviation (*P* <0.05).

* Significance level was calculated using 10, 000 randomization, 500 batches and 10,000 iterations (*P* < 0.01).

### Data analysis

#### Genotyping error and data validation

We genotyped each sample thrice to minimize genotyping errors and only consensus genotypes were processed for further analysis. Maximum likelihood allele dropout (ADO) and false allele (FA) error rates were quantified using PEDANT version 1.0 involving 10,000 search steps for enumeration of per allele error rates [33].

#### Scrutinizing microsatellites for individual identification

Identification of unique genotypes is primarily influenced by the selection of loci since the population can easily be under or over estimated depending on the select panel of loci. Therefore, for an unbiased estimation, we utilized three parameters in selection of microsatellites, (1) short amplicon size (assuming a relatively shorter amplicon will yield high amplification success with potentially degraded DNA samples); (2) having no or least genotyping errors and missing values (to avoid ambiguity in identification of unique genotypes and (3) an informative P_ID_ value (probability of obtaining identical genotypes between two samples by chance). Following these criteria, we scrutinized a panel of seven microsatellites and unique genotypes were identified using ALLELEMATCH package of R from the multi-locus genotype data [34]. The program finds the similarities between the samples using a metric of the Hamming distance [35] and uses hierarchical clustering and a dynamic method for identifying clusters on a dendrogram using the Dynamic Tree Cut package for R [36]. The locus wise and cumulative probability of identity for unrelated individuals (P_ID_) and siblings (P_ID_ sibs) was calculated using identity analysis module in GenAlEx version 6.5 [37].

#### Genetic polymorphism and extent of inbreeding

The per locus diversity was quantified by estimating the numbers of observed (N_a_) and effective alleles (N_e_), observed (H_o_) and expected (H_e_) heterozygosity using POPGENE version 1.32 [38]. The polymorphic information content (PIC), an indicator of marker’s informativeness and predicted null allele frequencies were calculated using CERVUS version 3.0 [39]. For the Hardy-Weinberg equilibrium test, we followed the probability test approach [40] using the program GENEPOP version 4.2 [41]. The unbiased estimator of Wright’s inbreeding coefficient (*F*_IS_) was calculated according to Weir and Cockerham [42] using GENEPOP version 4.2. Linkage disequilibrium (LD) was tested using GENEPOP version 4.2 to determine the extent of distortion from independent segregation of loci following 10,000 dememorizations, 500 batches and 10,000 iterations per batch [41].

#### Genetic relatedness and inference of population genetic structure

Average pairwise relatedness among free ranging black bears was calculated using Queller and Goodnight [43] relatedness coefficient (*r*) as implemented in GenAlEx version 6.5 [37]. The fractions of alleles shared among the individuals are determinant of relatedness coefficient value which ranges between −1 to +1. Principally, the *r* value is expected to be closer to 0.50 in first order relatives (full sibs and parent-offspring). The second order relatives (half siblings/grandparent-grant child) should exhibit an *r* value close to 0.25, followed by an *r* value close to 0.125 in the case of the third order relatives (first cousins). The *r* value below than 0.125 or a negative *r* value is likely to be an indicator of the unrelated individuals. The presence of population genetic structure was inferred using the Bayesian method as implemented in STRUCTURE version 2.3.3 [44]. We followed an admixture model and a model of correlated allele frequencies with burn‐in period of 5 ✕ 10^4^ and 5✕ 10^5^ Markov Chain Monte Carlo (MCMC) repetitions. Twenty independent replicates were run considering there were K populations (*K* = 1 to 10) without prior knowledge of sampling locations (NOPRIOR). Each individual was assigned to the inferred clusters using a threshold proportion of membership (q), *i*.*e*. q ≥0.80, else the individual was determined as admixed, if q < 0.80 [20, 45].

#### Genetic tagging and Land use/land cover utilization by translocated bears

The DNA extracts of the translocated bears were genotyped multiple times with the select panel of seven microsatellites to generate individual specific consensus genotypes. Each genotype was then assigned to a unique identifier (genetic tags) and their recaptures were subsequently tracked in the pool of genotypes generated from the samples collected from the hair snare stations placed in DNP and from the barb wire fencing deployed all around the horticulture croplands. The GPS coordinates of the genotypes of the translocated bears and their recaptures were plotted on map in GIS environment using ArcGIS version 10.0 (ESRI, CA, USA).

The information related to date of capture, release and site characteristics of translocated bears were used in understanding their activity centre and Land use/land cover (LULC) utilization patterns in the landscape. The composition analysis was performed following Aebischer et al. [46] and 50% & 95% kernel isopleths were generated for all 11 translocated bears using GME software [47] and available area for use was estimated by adding the area of activity estimated using 95% kernel isopleths. Landsat-5 satellite imagery (Spatial resolution = 30 m) downloaded from Global Land Cover Facility was classified at 1:50,000 scale using the program ERDAS Image version 9.0 (ERDAS, Inc, Atlanta, Georgia) [48]. The overall accuracy was 91.33% (S1 and S2 Tables). DL is composed of eight LULC classes *i*.*e*. mixed forest, pine forest, grassland & scrubland, human habitation, orchard, water, alpine and snow cover. We incorporated five LULC classes in analysis as the later three classes *i*.*e*. water, alpine and snow have not been utilized by black bears [1, 5]. All analysis pertaining to LULC composition and the proportion of locations of each animal within each LULC types was performed in ArcGIS version 10.0 (ESRI, CA, USA).

## Results

Three out of 20 loci (G10H, MSUT3 and UT23) did not amplify at all with 18 blood DNA extracts possibly because of the point mutation at primer binding sites of these loci. Out of 200 hair samples, 16 samples were few in numbers (<5 hairs per sample) to go for DNA extraction. Twenty four hair samples did not amplify for majority of the loci, possibly due to the low yield or poor quality of DNA extracts. Thus, we processed 160 hair samples for fragment analysis with 17 microsatellite loci. Twelve samples did not yield consensus genotypes after triplicate genotyping, hence were excluded from further analysis. Five loci *i*.*e*. UT3, UT25, MSUT5, UT38 and UT31 showed amplification success lower than 50% while remaining 12 loci yielded amplification success higher than 70%. Thus, 148 consensus genotypes with 12 microsatellites were used for genetic analysis.

### Screening of microsatellites with blood samples

All 17 microsatellite loci were found to be polymorphic and no locus showed apparent genotypic error with blood DNA extracts. The descriptive diversity measures are presented in Table 1. For individual identification, we scrutinized a panel of seven loci (MSUT2+ G10J+ MSUT8+ MSUT4+ UT1+ UT4+ UT35) out of 17 loci that yielded efficient cumulative P_ID_ sibs value *i*.*e*. P_ID_ sibs 0.001 (1 in 1000). The locus wise P_ID_ and P_ID_ sibs varied from 0.04–0.18 to 0.34–0.46, respectively. Altogether, 117 different alleles were found, ranging from 3 (UT36) to 10 (UT31), with an overall mean number of alleles per locus of 6.88 (± 0.45). The effective number of alleles ranged from 1.55 (MSUT1) to 8.17 (UT31) with a mean at 4.41 ± 0.43. The mean *H*o and *H*e were 0.68 ± 0.06 and 0.75 ± 0.04, respectively. All 17 loci except UT36 and MSUT1 exhibited PIC>0.5 and the average inbreeding coefficient was significantly different from zero (*F*_IS_ 0.11±0.07; *P* < 0.01). Ten out of the 17 loci followed HWE (*P >* 0.05) while seven loci (UT1, UT4, UT35, MSUT7, MSUT6, MSUT1 and MSUT5) deviated significantly from HWE. Six loci (UT1, MSUT7, UT36, MSUT6, MSUT1 and MSUT5) showed considerable proportion of null alleles.

### Individual identification and genetic polymorphism with hairs samples

**Fig 2:**
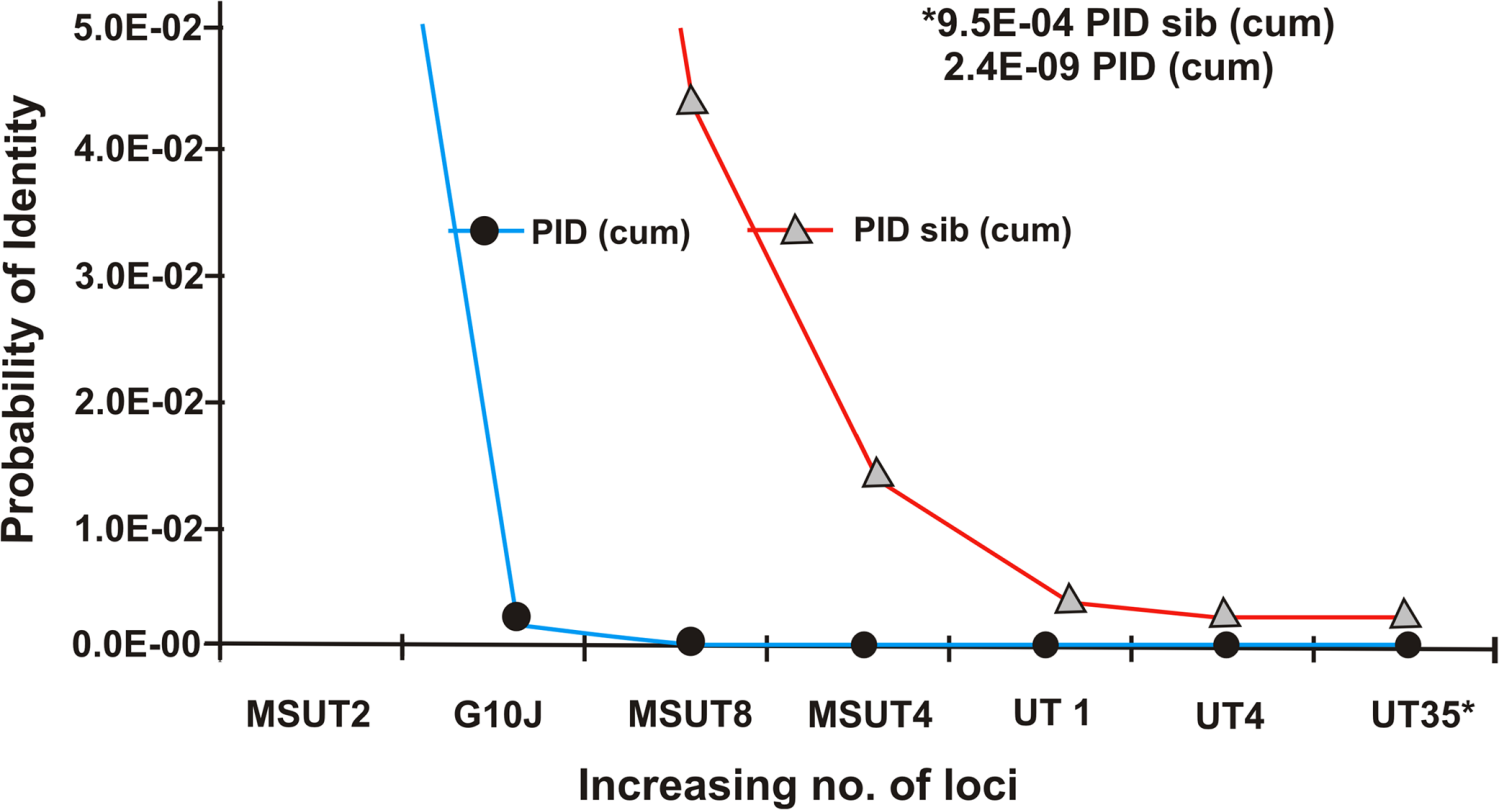
Select panel of seven polymorphic microsatellite loci used for individual identification of Asiatic black bears.

The observed ADO and FA error rates were not significant for any of the loci except for locus UT35. Using the select panel of seven loci, we identified 109 unique genotypes with few recaptures (S1 Fig and S3 Table). The estimated cumulative P_ID_ sibs of the select panel was 0.0009 (9 in 10000) (Fig 2). Locus wise estimated P_ID_ and P_ID_ sibs ranged from 0.01–0.2 to 0.30–0.51, respectively (Table 2). After individualization with select panel, we applied all 12 microsatellite data (with amplification success >70%) for enumeration of the genetic attributes of the Asiatic black bear population (Table 2). The wild caught samples showed sound genetic variation with a mean of 10.42 (±1.17) alleles, ranging from 5 (MSUT7) to 17 (G10J) alleles. The N_e_ ranged from 1.72 (UT36) to 9.57 (G10J) alleles with a mean of 4.59 (±0.72) alleles. The *H*o and *H*e ranged from 0.3 (UT36) to 0.97 (UT35) and from 0.42 (UT 36) and 0.90 (G10J), respectively. While heterozygote deficiency at locus MSUT6 (0.08) can be attributed to the low amplification success as there was about 30% missing data at this locus. The mean *H*o and *H*e were 0.58 ± 0.08 and 0.72 ± 0.04, respectively. Several loci *i*.*e*. UT1, MSUT7, UT29, UT36 and MSUT6 showed a moderate proportions of null alleles and 11 out of 12 loci deviated significantly from HWE (P<0.05) except for locus MSUT2. All loci showed PIC value higher than 0.5 except locus UT36 and the estimated average inbreeding coefficient was significantly different from zero (F_IS_ = 0.19 ± 0.10; *P* < 0.01).

**Table 2:** Genetic polymorphism of Asiatic black bear population at Dachigam landscape (N = 109 individuals)

Locus	Success rate	Na^1^	Ne^2^	Ho^3^	He^4^	PIC^5^	Fis^6^ (W&C)	P_ID_ ^7^ (locus)	P_ID_ sibs^8^ (locus)	ADO^9^	FA^10^	F_Null_ ^11^
MSUT2 ^H^	93.64	9	3.43	0.72	0.71	0.67	0.00	1.2E-01	4.2E-01	0.02	0.00	-0.01
G10J ^H^ †	95.45	17	9.57	0.78	0.90	0.89	0.14	1.9E-02	3.0E-01	0.13	0.00	0.07
MSUT8 ^H^ †	81.82	13	6.76	0.80	0.86	0.84	0.07	3.8E-02	3.3E-01	0.04	0.02	0.03
MSUT4 ^H^ †	96.36	14	7.74	0.87	0.88	0.86	0.01	3.1E-02	3.2E-01	0.16	0.02	0.00
UT1 ^H^ †	78.18	13	6.07	0.49	0.84	0.82	0.42	4.3E-02	3.4E-01	0.12	0.02	0.26
UT4 ^H^ †	90.00	8	4.48	0.70	0.78	0.75	0.10	8.1E-02	3.8E-01	0.03	0.04	0.05
UT35 ^H^ †	89.09	6	2.44	0.97	0.59	0.50	-0.64	2.5E-01	5.1E-01	0.70	0.00	-0.26
MSUT7†	74.55	5	2.70	0.38	0.63	0.57	0.40	2.0E-01	4.8E-01	0.15	0.03	0.24
UT29†	73.64	16	5.21	0.41	0.81	0.78	0.49	6.1E-02	3.6E-01	0.00	0.02	0.32
UT36†	76.36	10	1.72	0.31	0.42	0.40	0.26	3.5E-01	6.2E-01	0.02	0.06	0.16
MSUT6†	70.91	7	2.48	0.08	0.60	0.56	0.87	2.0E-01	5.0E-01	0.03	0.02	0.77
MSUT1†	93.64	7	2.43	0.47	0.59	0.52	0.20	2.4E-01	5.1E-01	0.08	0.02	0.08
Mean		10.42	4.59	0.58	0.72	0.68	0.19*					
SE		1.17	0.72	0.08	0.04	0.05	0.10					

1—observed number of alleles

2—effective number of alleles

3—observed heterozygosity

4—expected heterozygosity

5 polymorphic information content

6—inbreeding coefficient

7—probability of identity (locus)

8—probability of identity for sibs (locus)

9- allelic dropout rate

10- false allele rate

11—predicted frequencies of null alleles.

H—locus used for individual identification

† HWE deviation (*P* <0.05).

*Significance level was calculated using 10, 000 randomization, 500 batches and 10,000 iterations (*P* < 0.01).

### Genetic relatedness and population genetic structure

**Fig 3:**
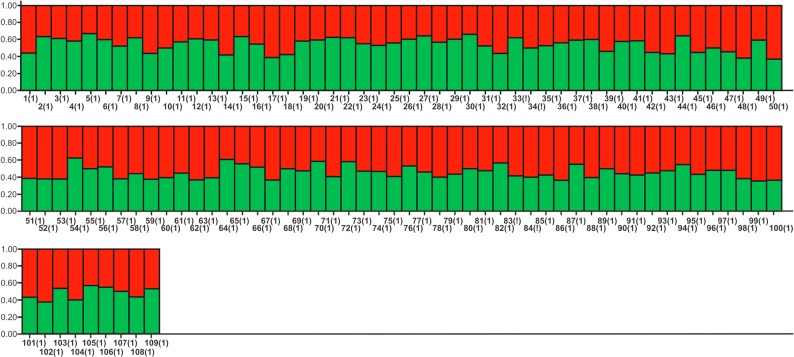
Bayesian clustering patterns of black bear population of Dachigam landscape.

The mean pairwise relatedness was found to be −0.012 (±0.003), indicating that analyzed samples largely belonged to unrelated individuals (detailed matrix of pairwise relatedness among individuals may be obtained upon request from the corresponding author). Eleven pairs of loci out of 66 pairwise comparisons (MSUT8-UT4, MSUT8-MSUT1, G10J-MSUT1, UT29-MSUT6, UT35-UT36, UT1-UT36, MSUT4-MSUT1, MSUT4-UT29, MSUT8-UT29, G10J-MSUT8, MSUT8-UT1) were in significant linkage disequilibrium (P<0.05). We excluded allelic data of 4 loci *i*.*e*. MSUT8, MSUT1, UT29 and UT36 that were in significant linkage disequilibrium with other loci following Pritchard et al. [44] which recommend using neutral markers lack in linkage disequilibrium for structure analysis. Thus, we employed 8 microsatellite data which lack in linkage disequilibrium for inferring population genetic structure. None of the individuals is assigned to any of the two clusters at K = 2 considering the threshold proportion of membership q ≥ 0.80 (Fig 3). Un-assignment of individuals was found to be consistent on increasing the number of clusters.

### Tracking the movement of translocated bears

**Fig 4:**
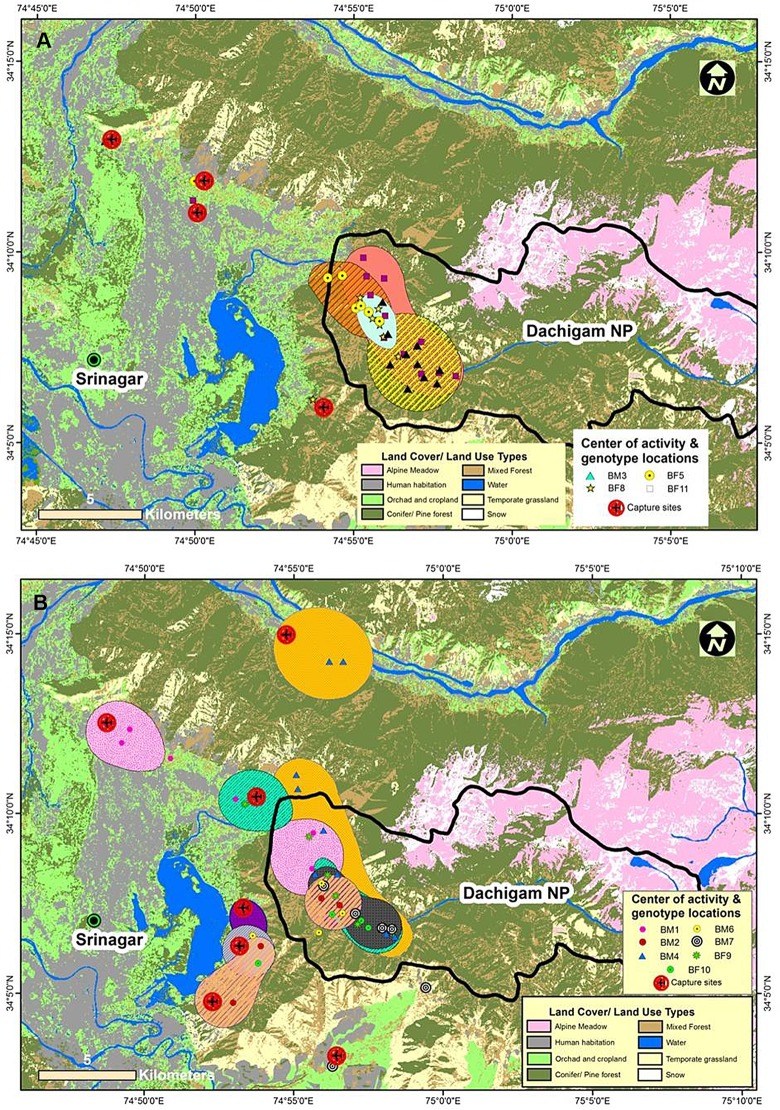
Map showing the capture sites of conflicting individuals and their center of activity over LULC classes in Dachigam landscape. (4a for bears that remained in DNP and 4b for bears which moved back to their capture sites after translocation).

Individual genetic profiles of 11 translocated bears and their number of recaptures are presented in Table 3. Out of 11 translocated bears captured from different conflict sites in DL, there were 6 males and 5 females, (henceforth male and female bears are represented as BM and BF, respectively). Genotypes of seven bears *i*.*e*. BM1, BM2, BM4, BM6, BM7, BF9 and BF10 were frequently re-captured away from the release sites. These animals thus explored fairly larger areas and moved across the landscape crossing forested habitats in DNP, croplands / orchards and human habitation in the landscape (Fig 4A). While the genotypes of four bears *i*.*e*. BM3, BF5, BF8 and BF11were recaptured among the samples collected within the close geographical proximity of their release sites (approx. within 5 km^2^; Fig 4B). These individuals, therefore, seemed to be settled down in DNP after translocation as their genotypes were not recaptured outside the boundary of DNP during the study period. The genotypes of wanderer bears were often recaptured close in proximity to their first physical capture. The locations of recaptures of these individuals indicated their repeated involvement in conflicts in the form of crop depredation and livestock killing in the landscape. The average days spent by the bears that returned to their capture sites after translocation were 10 days with a maximum stay of 15 days by bear BM4 and a minimum stay of 7 days by bear BM6 (S4 Table). Interestingly, five of seven bears that returned to their capture sites were translocated in spring and two in autumn. Four bears that remained in DNP, three were translocated in summer and one in autumn (S4 Table). Furthermore, out of the seven bears that returned to their capture sites, five were males while three of the four bears that settled in DNP were females.

**Table 3:** Molecular signature of 11 translocated black bears with the select panel of seven loci along with their number of recaptures.

Individual IDs	MSUT2^a^	G10J^b^	MSUT8^a^	MSUT4^a^	UT1^c^	UT4^c^	UT35^c^	Recaptures in the data
**BM1**	71/71	78/88	114/114	93/99	172/172	152/160	202/210	8
**BM2**	71/81	68/92	-99/-99†	87/101	176/212	152/164	201/210	5
**BM3**	71/71	64/64	-99/-99†	83/85	176/176	152/160	202/210	6
**BM4**	71/81	90/92	112/128	97/101	176/176	152/152	-99/-99†	11
**BF5**	-99/-99†	68/80	110/128	91/99	176/176	148/152	202/210	7
**BM6**	71/79	82/92	108/108	91/91	200/208	152/152	202/210	4
**BM7**	71/71	90/90	110/128	81/91	176/176	148/152	202/210	5
**BF8**	85/87	80/96	106/118	95/101	176/180	164/172	202/206	4
**BF9**	71/81	82/88	118/128	93/101	212/212	148/160	202/210	4
**BF10**	71/85	68/90	110/114	93/95	200/200	152/152	202/210	6
**BF11**	71/75	90/92	110/110	97/99	200/200	152/152	202/210	5

a—[31]

b—[21]

c—[30]

† missing values

### Spatial LULC of the translocated bears

In total, 65 locations of the translocated bears were recorded in DL. The overall land use between the bears that returned to their capture sites and bears that get settled in DNP differed significantly across various LULC types (λ = 0.0349, χ^2^ = 23.48, df = 5, P < 0.001, n = 7 and λ = 0.02835, χ^2^ = 19.60, df = 3, P < 0.001, n = 4). Use of different LULC types was disproportional to their availability within the area of analysis for all 11 translocated bears (Fig 4A and 4B). The mean area of activity of seven bears which returned to their capture sites at 50% isopleths (19.4 ± 6.0 km^2^) was larger than bears which stayed in DNP (9.7 ± 2.6 km^2^) (S5 and S6 Tables). Individuals that returned to their capture sites preferred cropland or orchards more than the other natural bear habitats. However, individuals that settled in DNP preferred natural forested LULC types. Compositional analysis of second order selection resulted in ranking matrices that ordered LULC types from the most to least use during the study period. For individuals which returned to their capture sites, the simplified matrix ranks the LULC type use in the order: Orchards/ croplands> Habitation> Mixed forest> Pine forest> Grassland and Scrubland. However, individuals that get settled in DNP, LULC type ranked in the order: Mixed forest> Pine forest> Grassland & scrubland> Orchards/ cropland> Habitation (S6 Table).

## Discussion

In this study, we established a molecular system for individual identification of free ranging Asiatic black bears using wild caught hair samples. The selected panel of seven microsatellites with cumulative P_ID_ sibs at 9.5 × 10^−4^ has proven to be more efficient in identifying individuals than reported in the previous studies conducted on bears (4.6 × 10^−3^ [49]; 9.14 × 10^−3^ [50]; 4.0 × 10^−3^ [51]; 2.15 × 10^−3^ [52]) and other mammals (5 × 10^−3^ [53]; 9 × 10^−3^) [25]. Thus, the select panel can be used for population estimation following capture-mark-recapture methods, assigning population genetic structure and investigating gene flow and other associated parameters. Majority of loci except locus MSUT2 deviated from HWE and this might have resulted by the occurrence of moderate proportions of null alleles at these loci (Table 2). Since, the Asiatic black bear population is large enough and sustains an adequate number of effect breeders thus we rule out the possibility to expect inbreeding in the population and a positive F_IS_ value is just an indication of homozygosity excess at majority of loci due to presence of moderate proportions of null alleles. The genetic assessment demonstrated that black bear population of DNP is flourishing and does not require any major management interventions in the present scenario.

Bayesian cluster analysis strongly indicated that the Asiatic black bear population inhabiting in DNP is under panmixia and lacks population genetic sub-structure. The capture and release of conflict bears to DNP from different sites in DL has been practiced by the Wildlife department, J&K since many years (perhaps in practice since more than one and half decade, no firm information available). Thus, we speculate that lack of population sub-structure might have contributed by the bears (often females) which remained in DNP after translocation and opportunistically breed with the resident bears. The results indicated that translocation success though questionable and needs to be revisited as many of the translocated bears returned to their capture sites but contributed fairly to keep the bear population under panmixia.

The movements of bears from protected areas to the human dominated landscape can be attributed with availability of high quality food outside in croplands than natural bear habitats of DNP during early springs and late autumn. The black bears in Taiwan were also reported to traverse large distances and negotiate human settlements to feed on selected food species [54]. Interestingly, five of the seven translocated bears who returned to their capture sites were males, while three of the four bears who remained in DNP were females. This indicated however with inclusion of a limited sample size that males were probably more exploratory and travel large areas while females were philopatric and perhaps preferred to remain in DNP after translocation. These speculations of bear movements especially large home range sizes of males than females and male’s extended travel to track the food resources in DNP were also supported by a long term study on studying ranging pattern of bears through radio-telemetry [5]. Since translocation was found to be more effective in summer when bear food was surplus in DNP whereas majority of bears, translocated to DNP in spring returned to their capture sites due to their uneasiness in the scarcity of food. Thus, results indicated that bear’s movement in DL is largely governed by the availability of food.

The larger activity area of bears that returned to their capture sites can be attributed with preference and selection of orchards and human habitation more than the availability in comparison to other land use type (S5 and S6 Tables). The food resources in DL vary spatio-temporary throughout the year. Spring and late autumn is identified as food stress time to DNP while summer harbors the surplus food resources. The easy access to food resources availability in summer inside DNP compels bears to get settled in DNP. Therefore, translocations carried out in summer reasonably succeeded over the translocation practiced in spring and late autumn. Due to low sample size, we could not determine sex effects on translocation success as majority of the bears translocated in autumn were males. But, we believe that season in terms of food availability seemed to be a major factor determining the translocation success of bears in DNP. The distribution of the food resources in the form of orchards and croplands are patchy outside DNP while they are homogenously distributed in the DNP [5]. This also revealed the causes of bear’s large distance and extended movement outside DNP to track food resources.

The current management practice of translocation of conflict bears to DNP without aversive conditioning laid by the Department of Wildlife Protection, J&K was found to be ineffective in mitigating bear-human conflicts on long term basis since, several bears returned to their capture sites. The returning of such bears to their capture sites can be attributed to retain sharp memory and the habituation to the high quality human food in crops and orchards. The studies conducted on the Asiatic black bear in similar complex topography in Japan and Taiwan with agricultural lands and developed areas immediately adjacent to the bear habitats also report exploitation of human food sources by bears [54, 55]. American black bears were also documented to return back to their capture sites from hundreds of kilometers due to the factors related to the reproductive behavior, mate selection, social structure, food resources availability and geographic barriers [11, 56, 57, 58]. Fairly large number of studies on bear documented that bears typically compete directly with humans for resources such as space, food, security and cover throughout their distributional ranges [4, 59, 60, 61, 62].

## Management Recommendations

All bear species are known to kill or injure livestock, damage agricultural or horticulture crops, or otherwise directly compete with humans in many areas [63]. The economic loss to locals arising due to crop and livestock depredation by bears is perpetuating particularly in Kashmir valley of J&K resulting in retaliatory killing of bears. This retaliatory approach of humans has become a major threat to bear population all through the Himalayan landscape in India in particular to Kashmir valley in Jammu and Kashmir [4]. Increased number of bear-human conflict cases might lead to development of antagonistic behavior in local communities which may decrease the efficacy of conservation efforts. For the long term survival and bear population management issues pertaining to bear-human conflicts in Kashmir and elsewhere need to be dealt consciously. Therefore, translocation of conflicting bears in DNP as an immediate remedy need to be revisited in the view of the fact that majority of the translocated bears had returned to their sites of capture. Since food resources availability plays a significant force for retaining bears in DNP, it is pertinent to mention that spatio-temporal mapping of bear food resources in DL will be useful for identifying potential areas where conflict bears can be released. None of the conflict bear at DL was aversively conditioned before translocation although several studies highlight that the aversive conditioning can reduce possibilities of animals to return back to their conflict sites [64, 65, 66, 67]. Hence, aversive conditioning in quarantine area might be attempted instead of an immediate translocation of conflict bears to DNP to enhance the possibility for bears to survive in the food scarcity conditions. Translocation of conflict bears to DNP carried out unknowingly can be more problematic since it will result in over augmentation and demographic changes which may aggravate rather mitigate conflict in the immediate vicinity of DNP. Additionally, conflict bears should be relocated to the nearest natural bear habitats in place of translocation into DNP.

## Supporting Information

S1 Fig Graph shows that an optimum for this data is allowing 2 alleles to mismatch (alleleMismatch = 2). (DOC)

S1 Table Different LULC classes in the study area. (DOC)

S2 Table Confusion matrices with respect to accuracy assessment of LULC classification in study area. (DOC)

S3 Table Allele match analysis for identification of unique genotypes. (PDF)

S4 Table Details of 11 translocated individual bears from elsewhere in the landscape to DNP. (DOC)

S5 Table Area of activity of bears which moved back to capture site and those which stayed in DNP after translocation based on 50% kernel isopleths. (DOC)

S6 Table Matrix of land use/land cover use rankings for Asiatic black bear based on comparing proportion of use within 95% isopleths with proportions of total available land use/ land cover types in the area of analysis in Dachigam landscape. (DOC)
