# Supplementary material for: Conflict Bear Translocation: Investigating Population Genetics and Fate of Bear Translocation in Dachigam National Park, Jammu and Kashmir, India
Source: PLoS One. 2015 Aug 12;10(8):e0132005. doi: 10.1371/journal.pone.0132005 (PMC4534036; doi:10.1371/journal.pone.0132005)
Supplement: S1 Fig — (DOC) [file pone.0132005.s001.doc]

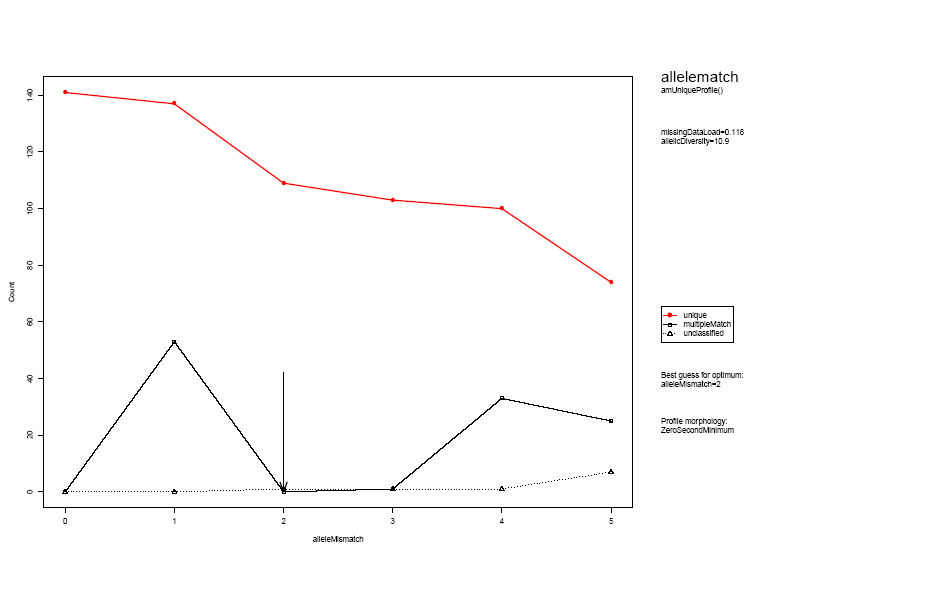


**S1 Fig. Graph shows that an optimum for this data is allowing 2 alleles to mismatch (alleleMismatch=2) as suggested by Galpern *et al.* (2012).**
