# Supplementary material for: Conflict Bear Translocation: Investigating Population Genetics and Fate of Bear Translocation in Dachigam National Park, Jammu and Kashmir, India
Source: PLoS One. 2015 Aug 12;10(8):e0132005. doi: 10.1371/journal.pone.0132005 (PMC4534036; doi:10.1371/journal.pone.0132005)
Supplement: S1 Table — (DOC) [file pone.0132005.s002.doc]

S1 Table. Different LULC classes in the study area

| **S. No.** | **LULC class** | **Area** | **% of total study area** |
| --- | --- | --- | --- |
| 1 | Conifer forest | 437 | 39.0 |
| 2 | Mixed forest | 146 | 13.5 |
| 3 | Temperate grassland and scrubland | 78 | 7.6 |
| 4 | Alpine Meadows | 88 | 8.2 |
| 5 | Human settlements | 145 | 13.1 |
| 6 | Orchards & Croplands | 139 | 12.2 |
| 7 | Snow | 35 | 3.2 |
| 8 | Water | 36 | 3.3 |
|  | Total | 1109 |  |

An area of 1109 km^2^ calculated using GIS was delineated for the distribution prediction model study considering the historic distribution of black bears. This area comprised of a mosaic of human habitation, crop lands, protected areas and territorial forest areas. Conifer forest account for maximum of 437 km^2^, followed by mixed forest (146 km^2^), human habitation (145km^2^), orchards & croplands (139km^2^) however snow cover and water accounts for about 35km^2^ and 36km^2^ respectively. (table1). The non-forest LULC includes human settlements, croplands, rocky clefts, water bodies and snow covered area. The spatial distribution of different land cover types was assessed after hybrid classification method. The Orchard and crop lands in the landscape largely represent horticulture and agriculture crops.
