# Supplementary material for: Conflict Bear Translocation: Investigating Population Genetics and Fate of Bear Translocation in Dachigam National Park, Jammu and Kashmir, India
Source: PLoS One. 2015 Aug 12;10(8):e0132005. doi: 10.1371/journal.pone.0132005 (PMC4534036; doi:10.1371/journal.pone.0132005)
Supplement: S2 Table — (DOC) [file pone.0132005.s003.doc]

**S2 Table. Confusion matrices with respect to accuracy assessment of LULC classification in study area**

| **Class name** | **Water** | **Conifer forest** | **Mixed forest** | **Orchard and cropland** | **Human habitation** | **Temperate grassland** | **Alpine meadow** | **Snow** | **Total** | **Users accuracy %** |
| --- | --- | --- | --- | --- | --- | --- | --- | --- | --- | --- |
| **Water** | 4 | 0 | 0 | 0 | 0 | 0 | 0 | 1 | 5 | 80.00% |
| **Conifer forest** | 0 | 63 | 2 | 0 | 0 | 1 | 1 | 1 | 68 | 92.65% |
| **Mixed forest** | 0 | 0 | 13 | 0 | 0 | 1 | 0 | 0 | 14 | 92.86% |
| **Orchard and cropland** | 0 | 1 | 2 | 17 | 0 | 0 | 0 | 0 | 20 | 85.00% |
| **Human habitation** | 1 | 0 | 1 | 0 | 10 | 0 | 0 | 0 | 12 | 83.33% |
| **Temperate grassland & scrubland** | 0 | 0 | 0 | 0 | 0 | 6 | 0 | 0 | 6 | 100.00% |
| **Alpine meadow** | 0 | 0 | 0 | 0 | 0 | 0 | 19 | 0 | 19 | 100.00% |
| **Snow** | 0 | 0 | 0 | 0 | 0 | 0 | 0 | 5 | 5 | 100.00% |
| **Total** | 5 | 64 | 18 | 17 | 10 | 8 | 20 | 7 | 149 |  |
| **Producer’s accuracy %** | 80.00% | 98.44% | 72.22% | 100.00% | 90.91% | 75.00% | 95.00% | 71.43% |  |  |

Overall Classification Accuracy = 91.33%

KAPPA (K^)

Overall Kappa Statistics = 0.8849

The classification accuracy of thematic map for LULC was derived from the error matrices listed in table 2. Overall classification accuracy was 91.33% however the lowest producer accuracy was observed for snow (71.43%) and mixed forest (72.22%). The overall Kappa’s statistics was 0.8849 indicating relatively good classification.
