# Supplementary material for: Conflict Bear Translocation: Investigating Population Genetics and Fate of Bear Translocation in Dachigam National Park, Jammu and Kashmir, India
Source: PLoS One. 2015 Aug 12;10(8):e0132005. doi: 10.1371/journal.pone.0132005 (PMC4534036; doi:10.1371/journal.pone.0132005)
Supplement: S3 Table — (PDF) [file pone.0132005.s004.pdf]

Supporting Information S3 table Allelematch analysis for identification of unique genotype

unique N=*109*
samples N=*148*

loci N=*7*
locus names: *MSUT.2a-MSUT.2b, G10Ja-G10Jb, MSUT.8a-MSUT.8b, MSUT.4a-MSUT.4b, UT.1a-UT.1b, UT.4a-UT.4b, UT.35a-UT.35b*

missing data represented by: *-99*
clustered genotypes consensus method: *1*
Psib calculated for: *samples with no mismatches*

alleleMismatch (m-hat; maximum number of mismatching alleles): *2*
cutHeight (d-hat; dynamic tree cutting height): *0.143*
matchThreshold (s-hat; lowest matching score returned): *0.857*

unclassified (samples that were not classified) N=*1*
multipleMatch (samples that match more than one unique genotype) N=*0*

Note: Unique genotypes are determined based on clustering of their scores followed by a dynamic tree cutting procedure (see supplementary documentation). *Psib appears for reference purposes and is not used to determine unique genotypes. It is calculated using allele frequencies in the unique genotype set.*

summary generated: *Tue Oct 15 00:30:29 2013*

Unique genotypes

		MSUT.2a	MSUT.2b	G10Ja	G10Jb	MSUT.8a	MSUT.8b	MSUT.4a	MSUT.4b	UT.1a	UT.1b	UT.4a	UT.4b	UT.35a	UT.35b	Psib	Type
Jump	MT-10	71	71	78	88	114	114	93	99	172	172	152	160	202	210	0.0013	UNIQUE
Jump	MT-11	71	81	68	92	-99	-99	87	101	176	212	152	164	202	210	0.0027	UNIQUE
Jump	MT-13	71	71	64	64	-99	-99	83	85	176	176	152	160	202	210	0.0039	UNIQUE
Jump	MT-131	71	81	90	92	112	128	97	101	176	176	152	152	-99	-99	0.0024	UNIQUE
Jump	MT-133	71	79	82	92	108	108	91	91	200	208	152	152	202	210	0.0011	UNIQUE
Jump	MT-134	-99	-99	68	80	110	128	91	99	176	176	148	152	202	210	0.0031	UNIQUE
Jump	MT-136	71	71	90	90	110	128	81	91	176	176	148	152	202	210	0.002	UNIQUE
Jump	MT-138	71	81	82	88	118	128	93	101	212	212	148	160	202	210	0.00078	UNIQUE
Jump	MT-139	-99	-99	-99	-99	86	86	93	99	176	176	148	152	202	210	0.0091	UNIQUE

Jump	MT-140	-99	-99	66	66	106	114	101	101	-99	-99	148	152	202	210	0.0052	UNIQUE
Jump	MT-141	71	85	68	90	110	114	93	95	200	200	152	152	202	210	0.0013	UNIQUE
Jump	MT-146	71	85	78	94	106	128	99	99	-99	-99	152	160	202	210	0.0031	UNIQUE
Jump	MT-154	71	75	90	92	110	110	97	99	200	208	152	152	202	210	0.0014	UNIQUE
Jump	MT-16	71	79	90	90	-99	-99	93	99	-99	-99	152	160	202	210	0.013	UNIQUE
Jump	MT-161	85	87	80	96	106	118	95	101	176	180	164	172	202	206	0.00031	UNIQUE
Jump	MT-169	71	85	88	94	110	122	87	101	176	176	148	156	202	206	0.00078	UNIQUE
Jump	MT-17	71	71	84	90	112	122	87	99	204	212	156	168	202	210	0.00072	UNIQUE
Jump	MT-170	71	71	82	92	110	120	93	93	208	208	148	160	202	206	0.00097	UNIQUE
Jump	MT-171	85	85	78	92	110	128	93	99	-99	-99	156	160	202	206	0.0019	UNIQUE
Jump	MT-172	79	91	92	96	110	128	95	101	180	180	160	164	202	206	0.00038	UNIQUE
Jump	MT-173	85	85	82	92	110	128	97	101	188	188	148	152	202	210	0.00083	UNIQUE
Jump	MT-175	71	73	64	90	110	110	97	99	176	184	-99	-99	202	210	0.003	UNIQUE
Jump	MT-178	71	85	76	94	120	128	99	99	-99	-99	152	160	202	210	0.0033	UNIQUE
Jump	MT-179	71	91	90	90	118	128	97	99	200	208	-99	-99	202	210	0.0026	UNIQUE
Jump	MT-18	71	79	80	86	108	118	95	97	168	176	148	160	202	214	0.00058	UNIQUE
Jump	MT-180	71	71	82	92	110	120	93	97	-99	-99	148	148	202	210	0.0039	UNIQUE
Jump	MT-182	71	85	94	96	118	120	95	97	212	212	148	152	202	210	0.001	UNIQUE
Jump	MT-184	91	91	90	96	110	114	93	99	196	204	160	160	202	210	0.00068	UNIQUE
Jump	MT-186	71	85	82	82	110	128	93	97	184	184	148	152	202	210	0.0012	UNIQUE
Jump	MT-187	71	75	90	92	114	118	93	99	168	168	152	160	202	210	0.001	UNIQUE
Jump	MT-188	85	91	96	96	108	114	93	95	204	212	156	160	206	210	0.00043	UNIQUE
Jump	MT-189	85	91	88	96	110	114	93	95	204	212	148	152	202	210	0.00085	UNIQUE
Jump	MT-192	71	87	88	94	114	128	97	101	212	212	148	172	202	210	0.00062	UNIQUE
Jump	MT-194	85	87	80	96	96	118	95	101	176	180	-99	-99	202	210	0.0016	UNIQUE
Jump	MT-195	85	91	90	96	118	122	93	99	172	208	168	176	202	210	0.00055	UNIQUE
Jump	MT-196	85	91	78	90	-99	-99	93	99	184	184	152	168	202	210	0.0021	UNIQUE
Jump	MT-198	75	75	88	92	-99	-99	93	99	180	180	152	160	202	210	0.0021	UNIQUE
Jump	MT-2	-99	-99	82	86	-99	-99	97	97	184	184	152	152	-99	-99	0.012	UNIQUE
Jump	MT-202	71	79	90	92	114	114	93	95	176	180	148	164	202	206	0.00075	UNIQUE
Jump	MT-203	79	85	90	96	108	118	93	97	176	176	152	156	202	210	0.0012	UNIQUE
Jump	MT-204	85	85	76	90	118	120	93	97	176	176	148	152	202	210	0.0014	UNIQUE
Jump	MT-207	71	85	76	90	110	120	87	95	176	184	152	152	202	210	0.0016	UNIQUE
Jump	MT-208	71	75	92	94	110	110	87	99	200	208	160	160	202	210	0.00096	UNIQUE

Jump	MT-209	75	85	76	76	-99	-99	91	97	208	208	148	152	202	210	0.0023	UNIQUE
Jump	MT-210	71	85	82	90	114	114	101	101	172	180	148	148	206	210	0.00064	UNIQUE
Jump	MT-212	71	71	82	92	110	120	87	93	-99	-99	148	148	206	210	0.0026	UNIQUE
Jump	MT-219	-99	-99	82	88	110	128	99	101	-99	-99	168	168	202	210	0.0054	UNIQUE
Jump	MT-22	71	85	90	90	-99	-99	93	97	-99	-99	152	160	-99	-99	0.027	UNIQUE
Jump	MT-223	85	85	64	88	108	108	95	97	-99	-99	-99	-99	202	210	0.007	UNIQUE
Jump	MT-226	71	85	82	88	108	108	87	101	-99	-99	-99	-99	202	210	0.0088	UNIQUE
Jump	MT-227	79	91	82	94	110	128	87	101	-99	-99	148	160	202	210	0.002	UNIQUE
Jump	MT-228	-99	-99	82	88	110	128	91	99	-99	-99	148	160	202	210	0.0073	UNIQUE
Jump	MT-25	71	85	88	94	110	122	87	99	-99	-99	148	156	202	210	0.0027	UNIQUE
Jump	MT-26	71	71	86	90	-99	-99	91	95	-99	-99	152	160	202	210	0.013	UNIQUE
Jump	MT-264	-99	-99	98	98	108	110	91	99	176	176	152	152	202	210	0.0041	UNIQUE
Jump	MT-28	71	71	82	84	110	114	89	93	-99	-99	148	156	-99	-99	0.0052	UNIQUE
Jump	MT-29	71	71	90	90	-99	-99	87	91	192	192	148	152	202	210	0.0037	UNIQUE
Jump	MT-31	71	85	90	96	-99	-99	87	99	-99	-99	152	160	202	206	0.0092	UNIQUE
Jump	MT-32	71	71	82	86	-99	-99	87	91	196	196	160	168	198	202	0.0015	UNIQUE
Jump	MT-33	71	71	88	90	-99	-99	93	97	204	204	152	160	202	210	0.0042	UNIQUE
Jump	MT-35	71	87	76	92	112	118	87	95	208	208	148	156	-99	-99	0.0011	UNIQUE
Jump	MT-36	71	75	76	90	-99	-99	-99	-99	208	208	160	160	-99	-99	0.015	UNIQUE
Jump	MT-39	71	79	84	90	118	122	-99	-99	176	200	168	168	202	210	0.0022	UNIQUE
Jump	MT-41	71	85	82	92	112	128	93	95	176	188	152	164	206	214	0.00051	UNIQUE
Jump	MT-43	71	81	90	94	-99	-99	93	95	-99	-99	160	160	202	210	0.0091	UNIQUE
Jump	MT-44	71	81	82	82	-99	-99	83	85	-99	-99	152	160	202	210	0.0074	UNIQUE
Jump	MT-46	71	81	78	90	-99	-99	87	93	-99	-99	-99	-99	202	210	0.023	UNIQUE
Jump	MT-47	71	71	90	90	-99	-99	93	93	-99	-99	160	168	202	210	0.013	UNIQUE
Jump	MT-48	71	71	76	92	-99	-99	91	93	168	208	164	164	202	210	0.0027	UNIQUE
Jump	MT-49	71	87	96	96	108	128	87	95	168	176	164	172	202	210	0.00064	UNIQUE
Jump	MT-5	71	79	92	94	122	122	83	85	-99	-99	156	168	-99	-99	0.0025	UNIQUE
Jump	MT-50	71	71	88	88	108	108	87	109	172	172	-99	-99	202	210	0.0028	UNIQUE
Jump	MT-51	71	71	92	92	100	108	-99	-99	172	172	152	160	202	210	0.004	UNIQUE
Jump	MT-52	71	85	80	90	108	114	-99	-99	176	176	160	164	202	210	0.0044	UNIQUE
Jump	MT-53	71	71	68	78	108	108	69	73	172	172	160	160	202	210	0.00086	UNIQUE
Jump	MT-54	-99	-99	-99	-99	-99	-99	91	99	176	176	-99	-99	202	210	0.081	UNIQUE
Jump	MT-55	71	75	76	76	108	108	87	91	176	176	152	152	202	210	0.0014	UNIQUE

Jump	MT-56	71	71	70	90	120	120	97	97	172	176	160	168	202	210	0.0011	UNIQUE
Jump	MT-57	71	79	96	98	108	108	95	95	176	176	164	168	202	210	0.00097	UNIQUE
Jump	MT-58	85	85	88	92	110	114	95	95	176	212	160	160	202	210	0.0012	UNIQUE
Jump	MT-59	71	85	86	92	110	118	87	93	180	208	152	164	202	210	0.0011	UNIQUE
Jump	MT-60	79	91	76	90	108	114	87	97	176	176	164	176	202	210	0.00073	UNIQUE
Jump	MT-62	71	85	88	92	110	114	93	93	172	172	160	164	202	210	0.0012	UNIQUE
Jump	MT-63	71	85	90	92	108	114	87	99	208	212	148	164	-99	-99	0.002	UNIQUE
Jump	MT-65	85	85	86	90	108	118	91	97	172	176	152	152	-99	-99	0.0023	UNIQUE
Jump	MT-66	71	79	86	90	108	120	91	99	180	204	164	168	202	210	0.00062	UNIQUE
Jump	MT-67	87	87	86	92	110	112	87	97	204	208	152	160	202	210	0.00065	UNIQUE
Jump	MT-68	71	87	78	92	108	118	91	97	176	208	152	160	202	210	0.0012	UNIQUE
Jump	MT-69	71	79	76	90	108	108	83	85	176	176	152	152	-99	-99	0.0024	UNIQUE
Jump	MT-70	85	87	84	90	108	110	91	95	176	176	160	160	202	202	0.0011	UNIQUE
Jump	MT-71	79	85	84	92	110	114	87	97	176	176	160	160	202	210	0.0012	UNIQUE
Jump	MT-72	71	85	90	96	118	128	91	95	172	212	156	168	202	206	0.00059	UNIQUE
Jump	MT-73	85	85	76	94	110	114	91	99	208	208	152	152	-99	-99	0.0019	UNIQUE
Jump	MT-74	71	85	80	90	108	110	93	99	176	212	152	156	234	234	0.00071	UNIQUE
Jump	MT-75	71	79	98	98	108	110	91	95	208	212	148	152	202	210	0.0011	UNIQUE
Jump	MT-76	75	85	78	98	112	128	91	97	176	212	152	152	202	210	0.00089	UNIQUE
Jump	MT-78	71	85	90	98	110	118	93	101	208	208	152	152	202	210	0.0016	UNIQUE
Jump	MT-82	71	71	-99	-99	100	130	69	89	172	212	160	160	202	210	0.0024	UNIQUE
Jump	MT-84	71	85	98	98	110	114	85	91	172	172	152	152	202	202	0.0012	UNIQUE
Jump	MT-85	71	91	78	98	110	112	93	97	-99	-99	152	156	202	206	0.0019	UNIQUE
Jump	MT-86	71	79	98	98	110	118	93	97	176	212	152	156	202	210	0.0012	UNIQUE
Jump	MT-87	79	85	98	98	114	120	93	97	156	200	148	152	202	210	0.0007	UNIQUE
Jump	MT-89	71	85	98	98	110	128	97	99	184	212	148	152	202	210	0.0012	UNIQUE
Jump	MT-90	71	91	92	98	114	120	91	91	176	200	148	152	202	206	0.0007	UNIQUE
Jump	MT-91	71	79	-99	-99	112	122	87	93	176	212	-99	-99	202	210	0.0084	UNIQUE
Jump	MT-92	79	85	88	98	110	110	87	95	176	212	148	152	202	210	0.0012	UNIQUE
Jump	MT-93	71	87	-99	-99	110	118	87	95	176	212	-99	-99	202	210	0.0096	UNIQUE
Jump	MT-94	79	85	74	74	110	114	97	97	172	172	-99	-99	-99	-99	0.0034	UNIQUE
Jump	MT-96	71	83	76	98	108	114	95	101	172	180	148	152	206	210	0.00055	UNIQUE

There were 109 unique genotypes identified using the parameters supplied.

Unclassified samples

		MSUT.2a	MSUT.2b	G10Ja	G10Jb	MSUT.8a	MSUT.8b	MSUT.4a	MSUT.4b	UT.1a	UT.1b	UT.4a	UT.4b	UT.35a	UT.35b	Type
!!!	MT-250	71	75	98	98	108	110	91	99	176	176	152	152	210	210	UNCLASSIFIED

There were 1 samples that were not classified using the parameters supplied.
Unclassified samples are often not sufficiently similar to match unique genotypes, and not sufficiently different to be declared unique themselves.
This is typically because of missing data. Please see the supplementary documentation for more information.

Unique genotype (1 of 109)

		MSUT.2a	MSUT.2b	G10Ja	G10Jb	MSUT.8a	MSUT.8b	MSUT.4a	MSUT.4b	UT.1a	UT.1b	UT.4a	UT.4b	UT.35a	UT.35b	Psib	Score	Type
	MT-10	71	71	78	88	114	114	93	99	172	172	152	160	202	210			UNIQUE
	MT-10	71	71	78	88	114	114	93	99	172	172	152	160	202	210	0.0013	1	MATCH
	MT-230	71	71	-99	-99	114	114	93	99	172	172	152	160	202	210	0.0048	0.93	MATCH
	MT-260	71	71	78	88	114	114	-99	-99	172	172	152	160	202	210	0.0039	0.93	MATCH

Unique genotype compared against 148 samples, returning those with score>=0.857
Psib is calculated for samples that have no mismatches. Differences among samples are due to loci with missing data.

Unique genotype (2 of 109)

		MSUT.2a	MSUT.2b	G10Ja	G10Jb	MSUT.8a	MSUT.8b	MSUT.4a	MSUT.4b	UT.1a	UT.1b	UT.4a	UT.4b	UT.35a	UT.35b	Psib	Score	Type
	MT-11	71	81	68	92	-99	-99	87	101	176	212	152	164	202	210			UNIQUE
	MT-11	71	81	68	92	-99	-99	87	101	176	212	152	164	202	210	0.0027	1	MATCH

Unique genotype compared against 148 samples, returning those with score>=0.857
Psib is calculated for samples that have no mismatches. Differences among samples are due to loci with missing data.

Unique genotype (3 of 109)

		MSUT.2a	MSUT.2b	G10Ja	G10Jb	MSUT.8a	MSUT.8b	MSUT.4a	MSUT.4b	UT.1a	UT.1b	UT.4a	UT.4b	UT.35a	UT.35b	Psib	Score	Type
	MT-13	71	71	64	64	-99	-99	83	85	176	176	152	160	202	210			UNIQUE
	MT-13	71	71	64	64	-99	-99	83	85	176	176	152	160	202	210	0.0039	1	MATCH

Unique genotype compared against 148 samples, returning those with score>=0.857
Psib is calculated for samples that have no mismatches. Differences among samples are due to loci with missing data.

Unique genotype (4 of 109)

		MSUT.2a	MSUT.2b	G10Ja	G10Jb	MSUT.8a	MSUT.8b	MSUT.4a	MSUT.4b	UT.1a	UT.1b	UT.4a	UT.4b	UT.35a	UT.35b	Psib	Score	Type

	MSUT.2a	MSUT.2b	G10Ja	G10Jb	MSUT.8a	MSUT.8b	MSUT.4a	MSUT.4b	UT.1a	UT.1b	UT.4a	UT.4b	UT.35a	UT.35b	Psib	Score	Type
MT-131	71	81	90	92	112	128	97	101	176	176	152	152	-99	-99			UNIQUE
MT-131	71	81	90	92	112	128	97	101	176	176	152	152	-99	-99	0.0024	1	MATCH

Unique genotype compared against 148 samples, returning those with score>=0.857
Psib is calculated for samples that have no mismatches. Differences among samples are due to loci with missing data.

Unique genotype (5 of 109)

	MSUT.2a	MSUT.2b	G10Ja	G10Jb	MSUT.8a	MSUT.8b	MSUT.4a	MSUT.4b	UT.1a	UT.1b	UT.4a	UT.4b	UT.35a	UT.35b	Psib	Score	Type
MT-133	71	79	82	92	108	108	91	91	200	208	152	152	202	210			UNIQUE
MT-133	71	79	82	92	108	108	91	91	200	208	152	152	202	210	0.0011	1	MATCH

Unique genotype compared against 148 samples, returning those with score>=0.857
Psib is calculated for samples that have no mismatches. Differences among samples are due to loci with missing data.

Unique genotype (6 of 109)

	MSUT.2a	MSUT.2b	G10Ja	G10Jb	MSUT.8a	MSUT.8b	MSUT.4a	MSUT.4b	UT.1a	UT.1b	UT.4a	UT.4b	UT.35a	UT.35b	Psib	Score	Type
MT-134	-99	-99	68	80	110	128	91	99	176	176	148	152	202	210			UNIQUE
MT-134	-99	-99	68	80	110	128	91	99	176	176	148	152	202	210	0.0031	1	MATCH

Unique genotype compared against 148 samples, returning those with score>=0.857
Psib is calculated for samples that have no mismatches. Differences among samples are due to loci with missing data.

Unique genotype (7 of 109)

	MSUT.2a	MSUT.2b	G10Ja	G10Jb	MSUT.8a	MSUT.8b	MSUT.4a	MSUT.4b	UT.1a	UT.1b	UT.4a	UT.4b	UT.35a	UT.35b	Psib	Score	Type
MT-136	71	71	90	90	110	128	81	91	176	176	148	152	202	210			UNIQUE
MT-136	71	71	90	90	110	128	81	91	176	176	148	152	202	210	0.002	1	MATCH

Unique genotype compared against 148 samples, returning those with score>=0.857
Psib is calculated for samples that have no mismatches. Differences among samples are due to loci with missing data.

Unique genotype (8 of 109)

	MSUT.2a	MSUT.2b	G10Ja	G10Jb	MSUT.8a	MSUT.8b	MSUT.4a	MSUT.4b	UT.1a	UT.1b	UT.4a	UT.4b	UT.35a	UT.35b	Psib	Score	Type
MT-138	71	81	82	88	118	128	93	101	212	212	148	160	202	210			UNIQUE
MT-138	71	81	82	88	118	128	93	101	212	212	148	160	202	210	0.00078	1	MATCH

Unique genotype compared against 148 samples, returning those with score>=0.857
Psib is calculated for samples that have no mismatches. Differences among samples are due to loci with missing data.

Unique genotype (9 of 109)

	MSUT.2a	MSUT.2b	G10Ja	G10Jb	MSUT.8a	MSUT.8b	MSUT.4a	MSUT.4b	UT.1a	UT.1b	UT.4a	UT.4b	UT.35a	UT.35b	Psib	Score	Type
MT-139	-99	-99	-99	-99	86	86	93	99	176	176	148	152	202	210			UNIQUE
MT-139	-99	-99	-99	-99	86	86	93	99	176	176	148	152	202	210	0.0091	1	MATCH

Unique genotype compared against 148 samples, returning those with score>=0.857
Psib is calculated for samples that have no mismatches. Differences among samples are due to loci with missing data.

Unique genotype (10 of 109)

	MSUT.2a	MSUT.2b	G10Ja	G10Jb	MSUT.8a	MSUT.8b	MSUT.4a	MSUT.4b	UT.1a	UT.1b	UT.4a	UT.4b	UT.35a	UT.35b	Psib	Score	Type
MT-140	-99	-99	66	66	106	114	101	101	-99	-99	148	152	202	210			UNIQUE
MT-140	-99	-99	66	66	106	114	101	101	-99	-99	148	152	202	210	0.0052	1	MATCH

Unique genotype compared against 148 samples, returning those with score>=0.857
Psib is calculated for samples that have no mismatches. Differences among samples are due to loci with missing data.

Unique genotype (11 of 109)

	MSUT.2a	MSUT.2b	G10Ja	G10Jb	MSUT.8a	MSUT.8b	MSUT.4a	MSUT.4b	UT.1a	UT.1b	UT.4a	UT.4b	UT.35a	UT.35b	Psib	Score	Type
MT-141	71	85	68	90	110	114	93	95	200	200	152	152	202	210			UNIQUE
MT-141	71	85	68	90	110	114	93	95	200	200	152	152	202	210	0.0013	1	MATCH

Unique genotype compared against 148 samples, returning those with score>=0.857
Psib is calculated for samples that have no mismatches. Differences among samples are due to loci with missing data.

Unique genotype (12 of 109)

	MSUT.2a	MSUT.2b	G10Ja	G10Jb	MSUT.8a	MSUT.8b	MSUT.4a	MSUT.4b	UT.1a	UT.1b	UT.4a	UT.4b	UT.35a	UT.35b	Psib	Score	Type
MT-146	71	85	78	94	106	128	99	99	-99	-99	152	160	202	210			UNIQUE
MT-146	71	85	78	94	106	128	99	99	-99	-99	152	160	202	210	0.0031	1	MATCH

Unique genotype compared against 148 samples, returning those with score>=0.857
Psib is calculated for samples that have no mismatches. Differences among samples are due to loci with missing data.

Unique genotype (13 of 109)

	MSUT.2a	MSUT.2b	G10Ja	G10Jb	MSUT.8a	MSUT.8b	MSUT.4a	MSUT.4b	UT.1a	UT.1b	UT.4a	UT.4b	UT.35a	UT.35b	Psib	Score	Type
MT-154	71	75	90	92	110	110	97	99	200	208	152	152	202	210			UNIQUE

MT-154	71	75	90	92	110	110	97	99	200	208	152	152	202	210	0.0014	1	MATCH

Unique genotype compared against 148 samples, returning those with score>=0.857
Psib is calculated for samples that have no mismatches. Differences among samples are due to loci with missing data.

Unique genotype (14 of 109)

	MSUT.2a	MSUT.2b	G10Ja	G10Jb	MSUT.8a	MSUT.8b	MSUT.4a	MSUT.4b	UT.1a	UT.1b	UT.4a	UT.4b	UT.35a	UT.35b	Psib	Score	Type
MT-16	71	79	90	90	-99	-99	93	99	-99	-99	152	160	202	210			UNIQUE
MT-16	71	79	90	90	-99	-99	93	99	-99	-99	152	160	202	210	0.013	1	MATCH

Unique genotype compared against 148 samples, returning those with score>=0.857
Psib is calculated for samples that have no mismatches. Differences among samples are due to loci with missing data.

Unique genotype (15 of 109)

	MSUT.2a	MSUT.2b	G10Ja	G10Jb	MSUT.8a	MSUT.8b	MSUT.4a	MSUT.4b	UT.1a	UT.1b	UT.4a	UT.4b	UT.35a	UT.35b	Psib	Score	Type
MT-161	85	87	80	96	106	118	95	101	176	180	164	172	202	206			UNIQUE
MT-161	85	87	80	96	106	118	95	101	176	180	164	172	202	206	0.00031	1	MATCH
MT-255	85	87	80	96	106	118	95	101	176	180	164	172	-99	-99	0.00077	0.93	MATCH

Unique genotype compared against 148 samples, returning those with score>=0.857
Psib is calculated for samples that have no mismatches. Differences among samples are due to loci with missing data.

Unique genotype (16 of 109)

	MSUT.2a	MSUT.2b	G10Ja	G10Jb	MSUT.8a	MSUT.8b	MSUT.4a	MSUT.4b	UT.1a	UT.1b	UT.4a	UT.4b	UT.35a	UT.35b	Psib	Score	Type
MT-169	71	85	88	94	110	122	87	101	176	176	148	156	202	206			UNIQUE
MT-169	71	85	88	94	110	122	87	101	176	176	148	156	202	206	0.00078	1	MATCH
MT-256	71	85	88	88	110	122	87	101	176	176	148	156	202	206	---	0.93	MATCH

Unique genotype compared against 148 samples, returning those with score>=0.857
Psib is calculated for samples that have no mismatches. Differences among samples are due to loci with missing data.

Unique genotype (17 of 109)

	MSUT.2a	MSUT.2b	G10Ja	G10Jb	MSUT.8a	MSUT.8b	MSUT.4a	MSUT.4b	UT.1a	UT.1b	UT.4a	UT.4b	UT.35a	UT.35b	Psib	Score	Type
MT-17	71	71	84	90	112	122	87	99	204	212	156	168	202	210			UNIQUE
MT-17	71	71	84	90	112	122	87	99	204	212	156	168	202	210	0.00072	1	MATCH
MT-231	71	71	84	90	112	122	97	99	204	212	156	168	202	210	---	0.93	MATCH

Unique genotype compared against 148 samples, returning those with score>=0.857
Psib is calculated for samples that have no mismatches. Differences among samples are due to loci with missing data.

Unique genotype (18 of 109)

	MSUT.2a	MSUT.2b	G10Ja	G10Jb	MSUT.8a	MSUT.8b	MSUT.4a	MSUT.4b	UT.1a	UT.1b	UT.4a	UT.4b	UT.35a	UT.35b	Psib	Score	Type
MT-170	71	71	82	92	110	120	93	93	208	208	148	160	202	206			UNIQUE
MT-170	71	71	82	92	110	120	93	93	208	208	148	160	202	206	0.00097	1	MATCH

Unique genotype compared against 148 samples, returning those with score>=0.857
Psib is calculated for samples that have no mismatches. Differences among samples are due to loci with missing data.

Unique genotype (19 of 109)

	MSUT.2a	MSUT.2b	G10Ja	G10Jb	MSUT.8a	MSUT.8b	MSUT.4a	MSUT.4b	UT.1a	UT.1b	UT.4a	UT.4b	UT.35a	UT.35b	Psib	Score	Type
MT-171	85	85	78	92	110	128	93	99	-99	-99	156	160	202	206			UNIQUE
MT-171	85	85	78	92	110	128	93	99	-99	-99	156	160	202	206	0.0019	1	MATCH

Unique genotype compared against 148 samples, returning those with score>=0.857
Psib is calculated for samples that have no mismatches. Differences among samples are due to loci with missing data.

Unique genotype (20 of 109)

	MSUT.2a	MSUT.2b	G10Ja	G10Jb	MSUT.8a	MSUT.8b	MSUT.4a	MSUT.4b	UT.1a	UT.1b	UT.4a	UT.4b	UT.35a	UT.35b	Psib	Score	Type
MT-172	79	91	92	96	110	128	95	101	180	180	160	164	202	206			UNIQUE
MT-172	79	91	92	96	110	128	95	101	180	180	160	164	202	206	0.00038	1	MATCH

Unique genotype compared against 148 samples, returning those with score>=0.857
Psib is calculated for samples that have no mismatches. Differences among samples are due to loci with missing data.

Unique genotype (21 of 109)

	MSUT.2a	MSUT.2b	G10Ja	G10Jb	MSUT.8a	MSUT.8b	MSUT.4a	MSUT.4b	UT.1a	UT.1b	UT.4a	UT.4b	UT.35a	UT.35b	Psib	Score	Type
MT-173	85	85	82	92	110	128	97	101	188	188	148	152	202	210			UNIQUE
MT-173	85	85	82	92	110	128	97	101	188	188	148	152	202	210	0.00083	1	MATCH
MT-257	85	85	82	92	110	128	97	97	188	188	148	152	202	210	---	0.93	MATCH

Unique genotype compared against 148 samples, returning those with score>=0.857
Psib is calculated for samples that have no mismatches. Differences among samples are due to loci with missing data.

Unique genotype (22 of 109)

	MSUT.2a	MSUT.2b	G10Ja	G10Jb	MSUT.8a	MSUT.8b	MSUT.4a	MSUT.4b	UT.1a	UT.1b	UT.4a	UT.4b	UT.35a	UT.35b	Psib	Score	Type
MT-175	71	73	64	90	110	110	97	99	176	184	-99	-99	202	210			UNIQUE
MT-175	71	73	64	90	110	110	97	99	176	184	-99	-99	202	210	0.003	1	MATCH
MT-258	71	71	64	90	110	110	97	99	176	184	-99	-99	202	210	---	0.93	MATCH
MT-266	71	73	64	90	110	110	-99	-99	176	184	-99	-99	202	210	0.0089	0.93	MATCH

Unique genotype compared against 148 samples, returning those with score>=0.857
Psib is calculated for samples that have no mismatches. Differences among samples are due to loci with missing data.

Unique genotype (23 of 109)

	MSUT.2a	MSUT.2b	G10Ja	G10Jb	MSUT.8a	MSUT.8b	MSUT.4a	MSUT.4b	UT.1a	UT.1b	UT.4a	UT.4b	UT.35a	UT.35b	Psib	Score	Type
MT-178	71	85	76	94	120	128	99	99	-99	-99	152	160	202	210			UNIQUE
MT-178	71	85	76	94	120	128	99	99	-99	-99	152	160	202	210	0.0033	1	MATCH

Unique genotype compared against 148 samples, returning those with score>=0.857
Psib is calculated for samples that have no mismatches. Differences among samples are due to loci with missing data.

Unique genotype (24 of 109)

	MSUT.2a	MSUT.2b	G10Ja	G10Jb	MSUT.8a	MSUT.8b	MSUT.4a	MSUT.4b	UT.1a	UT.1b	UT.4a	UT.4b	UT.35a	UT.35b	Psib	Score	Type
MT-179	71	91	90	90	118	128	97	99	200	208	-99	-99	202	210			UNIQUE
MT-179	71	91	90	90	118	128	97	99	200	208	-99	-99	202	210	0.0026	1	MATCH

Unique genotype compared against 148 samples, returning those with score>=0.857
Psib is calculated for samples that have no mismatches. Differences among samples are due to loci with missing data.

Unique genotype (25 of 109)

	MSUT.2a	MSUT.2b	G10Ja	G10Jb	MSUT.8a	MSUT.8b	MSUT.4a	MSUT.4b	UT.1a	UT.1b	UT.4a	UT.4b	UT.35a	UT.35b	Psib	Score	Type
MT-18	71	79	80	86	108	118	95	97	168	176	148	160	202	214			UNIQUE
MT-18	71	79	80	86	108	118	95	97	168	176	148	160	202	214	0.00058	1	MATCH
MT-232	71	79	80	86	108	118	95	97	168	176	148	160	202	214	0.00058	1	MATCH

Unique genotype compared against 148 samples, returning those with score>=0.857
Psib is calculated for samples that have no mismatches. Differences among samples are due to loci with missing data.

Unique genotype (26 of 109)

	MSUT.2a	MSUT.2b	G10Ja	G10Jb	MSUT.8a	MSUT.8b	MSUT.4a	MSUT.4b	UT.1a	UT.1b	UT.4a	UT.4b	UT.35a	UT.35b	Psib	Score	Type
MT-180	71	71	82	92	110	120	93	97	-99	-99	148	148	202	210			UNIQUE
MT-180	71	71	82	92	110	120	93	97	-99	-99	148	148	202	210	0.0039	1	MATCH

Unique genotype compared against 148 samples, returning those with score>=0.857
Psib is calculated for samples that have no mismatches. Differences among samples are due to loci with missing data.

Unique genotype (27 of 109)

	MSUT.2a	MSUT.2b	G10Ja	G10Jb	MSUT.8a	MSUT.8b	MSUT.4a	MSUT.4b	UT.1a	UT.1b	UT.4a	UT.4b	UT.35a	UT.35b	Psib	Score	Type
MT-182	71	85	94	96	118	120	95	97	212	212	148	152	202	210			UNIQUE
MT-182	71	85	94	96	118	120	95	97	212	212	148	152	202	210	0.001	1	MATCH

Unique genotype compared against 148 samples, returning those with score>=0.857
Psib is calculated for samples that have no mismatches. Differences among samples are due to loci with missing data.

Unique genotype (28 of 109)

	MSUT.2a	MSUT.2b	G10Ja	G10Jb	MSUT.8a	MSUT.8b	MSUT.4a	MSUT.4b	UT.1a	UT.1b	UT.4a	UT.4b	UT.35a	UT.35b	Psib	Score	Type
MT-184	91	91	90	96	110	114	93	99	196	204	160	160	202	210			UNIQUE
MT-184	91	91	90	96	110	114	93	99	196	204	160	160	202	210	0.00068	1	MATCH

Unique genotype compared against 148 samples, returning those with score>=0.857
Psib is calculated for samples that have no mismatches. Differences among samples are due to loci with missing data.

Unique genotype (29 of 109)

	MSUT.2a	MSUT.2b	G10Ja	G10Jb	MSUT.8a	MSUT.8b	MSUT.4a	MSUT.4b	UT.1a	UT.1b	UT.4a	UT.4b	UT.35a	UT.35b	Psib	Score	Type
MT-186	71	85	82	82	110	128	93	97	184	184	148	152	202	210			UNIQUE
MT-186	71	85	82	82	110	128	93	97	184	184	148	152	202	210	0.0012	1	MATCH

Unique genotype compared against 148 samples, returning those with score>=0.857
Psib is calculated for samples that have no mismatches. Differences among samples are due to loci with missing data.

Unique genotype (30 of 109)

	MSUT.2a	MSUT.2b	G10Ja	G10Jb	MSUT.8a	MSUT.8b	MSUT.4a	MSUT.4b	UT.1a	UT.1b	UT.4a	UT.4b	UT.35a	UT.35b	Psib	Score	Type
MT-187	71	75	90	92	114	118	93	99	168	168	152	160	202	210			UNIQUE
MT-187	71	75	90	92	114	118	93	99	168	168	152	160	202	210	0.001	1	MATCH
MT-259	71	75	90	92	114	114	93	99	168	168	152	160	202	210	---	0.93	MATCH

Unique genotype compared against 148 samples, returning those with score>=0.857
Psib is calculated for samples that have no mismatches. Differences among samples are due to loci with missing data.

Unique genotype (31 of 109)

	MSUT.2a	MSUT.2b	G10Ja	G10Jb	MSUT.8a	MSUT.8b	MSUT.4a	MSUT.4b	UT.1a	UT.1b	UT.4a	UT.4b	UT.35a	UT.35b	Psib	Score	Type
MT-188	85	91	96	96	108	114	93	95	204	212	156	160	206	210			UNIQUE
MT-188	85	91	96	96	108	114	93	95	204	212	156	160	206	210	0.00043	1	MATCH

Unique genotype compared against 148 samples, returning those with score>=0.857
Psib is calculated for samples that have no mismatches. Differences among samples are due to loci with missing data.

Unique genotype (32 of 109)

	MSUT.2a	MSUT.2b	G10Ja	G10Jb	MSUT.8a	MSUT.8b	MSUT.4a	MSUT.4b	UT.1a	UT.1b	UT.4a	UT.4b	UT.35a	UT.35b	Psib	Score	Type
MT-189	85	91	88	96	110	114	93	95	204	212	148	152	202	210			UNIQUE
MT-189	85	91	88	96	110	114	93	95	204	212	148	152	202	210	0.00085	1	MATCH

Unique genotype compared against 148 samples, returning those with score>=0.857
Psib is calculated for samples that have no mismatches. Differences among samples are due to loci with missing data.

Unique genotype (33 of 109)

	MSUT.2a	MSUT.2b	G10Ja	G10Jb	MSUT.8a	MSUT.8b	MSUT.4a	MSUT.4b	UT.1a	UT.1b	UT.4a	UT.4b	UT.35a	UT.35b	Psib	Score	Type
MT-192	71	87	88	94	114	128	97	101	212	212	148	172	202	210			UNIQUE
MT-192	71	87	88	94	114	128	97	101	212	212	148	172	202	210	0.00062	1	MATCH

Unique genotype compared against 148 samples, returning those with score>=0.857
Psib is calculated for samples that have no mismatches. Differences among samples are due to loci with missing data.

Unique genotype (34 of 109)

	MSUT.2a	MSUT.2b	G10Ja	G10Jb	MSUT.8a	MSUT.8b	MSUT.4a	MSUT.4b	UT.1a	UT.1b	UT.4a	UT.4b	UT.35a	UT.35b	Psib	Score	Type
MT-194	85	87	80	96	96	118	95	101	176	180	-99	-99	202	210			UNIQUE
MT-194	85	87	80	96	96	118	95	101	176	180	-99	-99	202	210	0.0016	1	MATCH

Unique genotype compared against 148 samples, returning those with score>=0.857
Psib is calculated for samples that have no mismatches. Differences among samples are due to loci with missing data.

Unique genotype (35 of 109)

	MSUT.2a	MSUT.2b	G10Ja	G10Jb	MSUT.8a	MSUT.8b	MSUT.4a	MSUT.4b	UT.1a	UT.1b	UT.4a	UT.4b	UT.35a	UT.35b	Psib	Score	Type
MT-195	85	91	90	96	118	122	93	99	172	208	168	176	202	210			UNIQUE
MT-195	85	91	90	96	118	122	93	99	172	208	168	176	202	210	0.00055	1	MATCH

Unique genotype compared against 148 samples, returning those with score>=0.857
Psib is calculated for samples that have no mismatches. Differences among samples are due to loci with missing data.

Unique genotype (36 of 109)

	MSUT.2a	MSUT.2b	G10Ja	G10Jb	MSUT.8a	MSUT.8b	MSUT.4a	MSUT.4b	UT.1a	UT.1b	UT.4a	UT.4b	UT.35a	UT.35b	Psib	Score	Type
MT-196	85	91	78	90	-99	-99	93	99	184	184	152	168	202	210			UNIQUE
MT-196	85	91	78	90	-99	-99	93	99	184	184	152	168	202	210	0.0021	1	MATCH

Unique genotype compared against 148 samples, returning those with score>=0.857
Psib is calculated for samples that have no mismatches. Differences among samples are due to loci with missing data.

Unique genotype (37 of 109)

	MSUT.2a	MSUT.2b	G10Ja	G10Jb	MSUT.8a	MSUT.8b	MSUT.4a	MSUT.4b	UT.1a	UT.1b	UT.4a	UT.4b	UT.35a	UT.35b	Psib	Score	Type
MT-198	75	75	88	92	-99	-99	93	99	180	180	152	160	202	210			UNIQUE
MT-198	75	75	88	92	-99	-99	93	99	180	180	152	160	202	210	0.0021	1	MATCH

Unique genotype compared against 148 samples, returning those with score>=0.857
Psib is calculated for samples that have no mismatches. Differences among samples are due to loci with missing data.

Unique genotype (38 of 109)

	MSUT.2a	MSUT.2b	G10Ja	G10Jb	MSUT.8a	MSUT.8b	MSUT.4a	MSUT.4b	UT.1a	UT.1b	UT.4a	UT.4b	UT.35a	UT.35b	Psib	Score	Type
MT-2	-99	-99	82	86	-99	-99	97	97	184	184	152	152	-99	-99			UNIQUE
MT-2	-99	-99	82	86	-99	-99	97	97	184	184	152	152	-99	-99	0.012	1	MATCH

Unique genotype compared against 148 samples, returning those with score>=0.857
Psib is calculated for samples that have no mismatches. Differences among samples are due to loci with missing data.

Unique genotype (39 of 109)

	MSUT.2a	MSUT.2b	G10Ja	G10Jb	MSUT.8a	MSUT.8b	MSUT.4a	MSUT.4b	UT.1a	UT.1b	UT.4a	UT.4b	UT.35a	UT.35b	Psib	Score	Type
MT-202	71	79	90	92	114	114	93	95	176	180	148	164	202	206			UNIQUE
MT-202	71	79	90	92	114	114	93	95	176	180	148	164	202	206	0.00075	1	MATCH

Unique genotype compared against 148 samples, returning those with score>=0.857
Psib is calculated for samples that have no mismatches. Differences among samples are due to loci with missing data.

Unique genotype (40 of 109)

	MSUT.2a	MSUT.2b	G10Ja	G10Jb	MSUT.8a	MSUT.8b	MSUT.4a	MSUT.4b	UT.1a	UT.1b	UT.4a	UT.4b	UT.35a	UT.35b	Psib	Score	Type
MT-203	79	85	90	96	108	118	93	97	176	176	152	156	202	210			UNIQUE
MT-203	79	85	90	96	108	118	93	97	176	176	152	156	202	210	0.0012	1	MATCH

Unique genotype compared against 148 samples, returning those with score>=0.857
Psib is calculated for samples that have no mismatches. Differences among samples are due to loci with missing data.

Unique genotype (41 of 109)

	MSUT.2a	MSUT.2b	G10Ja	G10Jb	MSUT.8a	MSUT.8b	MSUT.4a	MSUT.4b	UT.1a	UT.1b	UT.4a	UT.4b	UT.35a	UT.35b	Psib	Score	Type
MT-204	85	85	76	90	118	120	93	97	176	176	148	152	202	210			UNIQUE
MT-204	85	85	76	90	118	120	93	97	176	176	148	152	202	210	0.0014	1	MATCH

Unique genotype compared against 148 samples, returning those with score>=0.857
Psib is calculated for samples that have no mismatches. Differences among samples are due to loci with missing data.

Unique genotype (42 of 109)

	MSUT.2a	MSUT.2b	G10Ja	G10Jb	MSUT.8a	MSUT.8b	MSUT.4a	MSUT.4b	UT.1a	UT.1b	UT.4a	UT.4b	UT.35a	UT.35b	Psib	Score	Type
MT-207	71	85	76	90	110	120	87	95	176	184	152	152	202	210			UNIQUE
MT-207	71	85	76	90	110	120	87	95	176	184	152	152	202	210	0.0016	1	MATCH

Unique genotype compared against 148 samples, returning those with score>=0.857
Psib is calculated for samples that have no mismatches. Differences among samples are due to loci with missing data.

Unique genotype (43 of 109)

	MSUT.2a	MSUT.2b	G10Ja	G10Jb	MSUT.8a	MSUT.8b	MSUT.4a	MSUT.4b	UT.1a	UT.1b	UT.4a	UT.4b	UT.35a	UT.35b	Psib	Score	Type
MT-208	71	75	92	94	110	110	87	99	200	208	160	160	202	210			UNIQUE
MT-208	71	75	92	94	110	110	87	99	200	208	160	160	202	210	0.00096	1	MATCH

Unique genotype compared against 148 samples, returning those with score>=0.857
Psib is calculated for samples that have no mismatches. Differences among samples are due to loci with missing data.

Unique genotype (44 of 109)

	MSUT.2a	MSUT.2b	G10Ja	G10Jb	MSUT.8a	MSUT.8b	MSUT.4a	MSUT.4b	UT.1a	UT.1b	UT.4a	UT.4b	UT.35a	UT.35b	Psib	Score	Type
MT-209	75	85	76	76	-99	-99	91	97	208	208	148	152	202	210			UNIQUE

	MSUT.2a	MSUT.2b	G10Ja	G10Jb	MSUT.8a	MSUT.8b	MSUT.4a	MSUT.4b	UT.1a	UT.1b	UT.4a	UT.4b	UT.35a	UT.35b	Psib	Score	Type
MT-209	75	85	76	76	-99	-99	91	97	208	208	148	152	202	210	0.0023	1	MATCH

Unique genotype compared against 148 samples, returning those with score>=0.857
Psib is calculated for samples that have no mismatches. Differences among samples are due to loci with missing data.

Unique genotype (45 of 109)

	MSUT.2a	MSUT.2b	G10Ja	G10Jb	MSUT.8a	MSUT.8b	MSUT.4a	MSUT.4b	UT.1a	UT.1b	UT.4a	UT.4b	UT.35a	UT.35b	Psib	Score	Type
MT-210	71	85	82	90	114	114	101	101	172	180	148	148	206	210			UNIQUE
MT-210	71	85	82	90	114	114	101	101	172	180	148	148	206	210	0.00064	1	MATCH

Unique genotype compared against 148 samples, returning those with score>=0.857
Psib is calculated for samples that have no mismatches. Differences among samples are due to loci with missing data.

Unique genotype (46 of 109)

	MSUT.2a	MSUT.2b	G10Ja	G10Jb	MSUT.8a	MSUT.8b	MSUT.4a	MSUT.4b	UT.1a	UT.1b	UT.4a	UT.4b	UT.35a	UT.35b	Psib	Score	Type
MT-212	71	71	82	92	110	120	87	93	-99	-99	148	148	206	210			UNIQUE
MT-212	71	71	82	92	110	120	87	93	-99	-99	148	148	206	210	0.0026	1	MATCH
MT-222	71	71	82	92	110	120	87	93	-99	-99	148	148	**202**	210	---	0.93	MATCH

Unique genotype compared against 148 samples, returning those with score>=0.857
Psib is calculated for samples that have no mismatches. Differences among samples are due to loci with missing data.

Unique genotype (47 of 109)

	MSUT.2a	MSUT.2b	G10Ja	G10Jb	MSUT.8a	MSUT.8b	MSUT.4a	MSUT.4b	UT.1a	UT.1b	UT.4a	UT.4b	UT.35a	UT.35b	Psib	Score	Type
MT-219	-99	-99	82	88	110	128	99	101	-99	-99	168	168	202	210			UNIQUE
MT-219	-99	-99	82	88	110	128	99	101	-99	-99	168	168	202	210	0.0054	1	MATCH

Unique genotype compared against 148 samples, returning those with score>=0.857
Psib is calculated for samples that have no mismatches. Differences among samples are due to loci with missing data.

Unique genotype (48 of 109)

	MSUT.2a	MSUT.2b	G10Ja	G10Jb	MSUT.8a	MSUT.8b	MSUT.4a	MSUT.4b	UT.1a	UT.1b	UT.4a	UT.4b	UT.35a	UT.35b	Psib	Score	Type
MT-22	71	85	90	90	-99	-99	93	97	-99	-99	152	160	-99	-99			UNIQUE
MT-22	71	85	90	90	-99	-99	93	97	-99	-99	152	160	-99	-99	0.027	1	MATCH

Unique genotype compared against 148 samples, returning those with score>=0.857
Psib is calculated for samples that have no mismatches. Differences among samples are due to loci with missing data.

Unique genotype (49 of 109)

	MSUT.2a	MSUT.2b	G10Ja	G10Jb	MSUT.8a	MSUT.8b	MSUT.4a	MSUT.4b	UT.1a	UT.1b	UT.4a	UT.4b	UT.35a	UT.35b	Psib	Score	Type
MT-223	85	85	64	88	108	108	95	97	-99	-99	-99	-99	202	210			UNIQUE
MT-223	85	85	64	88	108	108	95	97	-99	-99	-99	-99	202	210	0.007	1	MATCH

Unique genotype compared against 148 samples, returning those with score>=0.857
Psib is calculated for samples that have no mismatches. Differences among samples are due to loci with missing data.

Unique genotype (50 of 109)

	MSUT.2a	MSUT.2b	G10Ja	G10Jb	MSUT.8a	MSUT.8b	MSUT.4a	MSUT.4b	UT.1a	UT.1b	UT.4a	UT.4b	UT.35a	UT.35b	Psib	Score	Type
MT-226	71	85	82	88	108	108	87	101	-99	-99	-99	-99	202	210			UNIQUE
MT-226	71	85	82	88	108	108	87	101	-99	-99	-99	-99	202	210	0.0088	1	MATCH

Unique genotype compared against 148 samples, returning those with score>=0.857
Psib is calculated for samples that have no mismatches. Differences among samples are due to loci with missing data.

Unique genotype (51 of 109)

	MSUT.2a	MSUT.2b	G10Ja	G10Jb	MSUT.8a	MSUT.8b	MSUT.4a	MSUT.4b	UT.1a	UT.1b	UT.4a	UT.4b	UT.35a	UT.35b	Psib	Score	Type
MT-227	79	91	82	94	110	128	87	101	-99	-99	148	160	202	210			UNIQUE
MT-227	79	91	82	94	110	128	87	101	-99	-99	148	160	202	210	0.002	1	MATCH

Unique genotype compared against 148 samples, returning those with score>=0.857
Psib is calculated for samples that have no mismatches. Differences among samples are due to loci with missing data.

Unique genotype (52 of 109)

	MSUT.2a	MSUT.2b	G10Ja	G10Jb	MSUT.8a	MSUT.8b	MSUT.4a	MSUT.4b	UT.1a	UT.1b	UT.4a	UT.4b	UT.35a	UT.35b	Psib	Score	Type
MT-228	-99	-99	82	88	110	128	91	99	-99	-99	148	160	202	210			UNIQUE
MT-228	-99	-99	82	88	110	128	91	99	-99	-99	148	160	202	210	0.0073	1	MATCH

Unique genotype compared against 148 samples, returning those with score>=0.857
Psib is calculated for samples that have no mismatches. Differences among samples are due to loci with missing data.

Unique genotype (53 of 109)

	MSUT.2a	MSUT.2b	G10Ja	G10Jb	MSUT.8a	MSUT.8b	MSUT.4a	MSUT.4b	UT.1a	UT.1b	UT.4a	UT.4b	UT.35a	UT.35b	Psib	Score	Type
MT-25	71	85	88	94	110	122	87	99	-99	-99	148	156	202	210			UNIQUE

MT-25	71	85	88	94	110	122	87	99	-99	-99	148	156	202	210	0.0027	1	MATCH

Unique genotype compared against 148 samples, returning those with score>=0.857
Psib is calculated for samples that have no mismatches. Differences among samples are due to loci with missing data.

Unique genotype (54 of 109)

	MSUT.2a	MSUT.2b	G10Ja	G10Jb	MSUT.8a	MSUT.8b	MSUT.4a	MSUT.4b	UT.1a	UT.1b	UT.4a	UT.4b	UT.35a	UT.35b	Psib	Score	Type
MT-26	71	71	86	90	-99	-99	91	95	-99	-99	152	160	202	210			UNIQUE
MT-26	71	71	86	90	-99	-99	91	95	-99	-99	152	160	202	210	0.013	1	MATCH

Unique genotype compared against 148 samples, returning those with score>=0.857
Psib is calculated for samples that have no mismatches. Differences among samples are due to loci with missing data.

Unique genotype (55 of 109)

	MSUT.2a	MSUT.2b	G10Ja	G10Jb	MSUT.8a	MSUT.8b	MSUT.4a	MSUT.4b	UT.1a	UT.1b	UT.4a	UT.4b	UT.35a	UT.35b	Psib	Score	Type
MT-264	-99	-99	98	98	108	110	91	99	176	176	152	152	202	210			UNIQUE
MT-264	-99	-99	98	98	108	110	91	99	176	176	152	152	202	210	0.0041	1	MATCH
MT-77	71	75	98	98	108	110	91	99	176	176	152	152	202	210	0.0041	0.93	MATCH

Unique genotype compared against 148 samples, returning those with score>=0.857
Psib is calculated for samples that have no mismatches. Differences among samples are due to loci with missing data.

Unique genotype (56 of 109)

	MSUT.2a	MSUT.2b	G10Ja	G10Jb	MSUT.8a	MSUT.8b	MSUT.4a	MSUT.4b	UT.1a	UT.1b	UT.4a	UT.4b	UT.35a	UT.35b	Psib	Score	Type
MT-28	71	71	82	84	110	114	89	93	-99	-99	148	156	-99	-99			UNIQUE
MT-28	71	71	82	84	110	114	89	93	-99	-99	148	156	-99	-99	0.0052	1	MATCH

Unique genotype compared against 148 samples, returning those with score>=0.857
Psib is calculated for samples that have no mismatches. Differences among samples are due to loci with missing data.

Unique genotype (57 of 109)

	MSUT.2a	MSUT.2b	G10Ja	G10Jb	MSUT.8a	MSUT.8b	MSUT.4a	MSUT.4b	UT.1a	UT.1b	UT.4a	UT.4b	UT.35a	UT.35b	Psib	Score	Type
MT-29	71	71	90	90	-99	-99	87	91	192	192	148	152	202	210			UNIQUE
MT-29	71	71	90	90	-99	-99	87	91	192	192	148	152	202	210	0.0037	1	MATCH
MT-233	71	71	90	90	-99	-99	87	91	192	192	148	152	202	210	0.0037	1	MATCH

Unique genotype compared against 148 samples, returning those with score>=0.857
Psib is calculated for samples that have no mismatches. Differences among samples are due to loci with missing data.

Unique genotype (58 of 109)

	MSUT.2a	MSUT.2b	G10Ja	G10Jb	MSUT.8a	MSUT.8b	MSUT.4a	MSUT.4b	UT.1a	UT.1b	UT.4a	UT.4b	UT.35a	UT.35b	Psib	Score	Type
MT-31	71	85	90	96	-99	-99	87	99	-99	-99	152	160	202	206			UNIQUE
MT-31	71	85	90	96	-99	-99	87	99	-99	-99	152	160	202	206	0.0092	1	MATCH
MT-234	71	85	90	96	-99	-99	87	99	-99	-99	152	152	202	206	---	0.93	MATCH

Unique genotype compared against 148 samples, returning those with score>=0.857
Psib is calculated for samples that have no mismatches. Differences among samples are due to loci with missing data.

Unique genotype (59 of 109)

	MSUT.2a	MSUT.2b	G10Ja	G10Jb	MSUT.8a	MSUT.8b	MSUT.4a	MSUT.4b	UT.1a	UT.1b	UT.4a	UT.4b	UT.35a	UT.35b	Psib	Score	Type
MT-32	71	71	82	86	-99	-99	87	91	196	196	160	168	198	202			UNIQUE
MT-32	71	71	82	86	-99	-99	87	91	196	196	160	168	198	202	0.0015	1	MATCH

Unique genotype compared against 148 samples, returning those with score>=0.857
Psib is calculated for samples that have no mismatches. Differences among samples are due to loci with missing data.

Unique genotype (60 of 109)

	MSUT.2a	MSUT.2b	G10Ja	G10Jb	MSUT.8a	MSUT.8b	MSUT.4a	MSUT.4b	UT.1a	UT.1b	UT.4a	UT.4b	UT.35a	UT.35b	Psib	Score	Type
MT-33	71	71	88	90	-99	-99	93	97	204	204	152	160	202	210			UNIQUE
MT-33	71	71	88	90	-99	-99	93	97	204	204	152	160	202	210	0.0042	1	MATCH

Unique genotype compared against 148 samples, returning those with score>=0.857
Psib is calculated for samples that have no mismatches. Differences among samples are due to loci with missing data.

Unique genotype (61 of 109)

	MSUT.2a	MSUT.2b	G10Ja	G10Jb	MSUT.8a	MSUT.8b	MSUT.4a	MSUT.4b	UT.1a	UT.1b	UT.4a	UT.4b	UT.35a	UT.35b	Psib	Score	Type
MT-35	71	87	76	92	112	118	87	95	208	208	148	156	-99	-99			UNIQUE
MT-35	71	87	76	92	112	118	87	95	208	208	148	156	-99	-99	0.0011	1	MATCH

Unique genotype compared against 148 samples, returning those with score>=0.857
Psib is calculated for samples that have no mismatches. Differences among samples are due to loci with missing data.

Unique genotype (62 of 109)

	MSUT.2a	MSUT.2b	G10Ja	G10Jb	MSUT.8a	MSUT.8b	MSUT.4a	MSUT.4b	UT.1a	UT.1b	UT.4a	UT.4b	UT.35a	UT.35b	Psib	Score	Type

MT-36	71	75	76	90	-99	-99	-99	-99	208	208	160	160	-99	-99			UNIQUE
MT-36	71	75	76	90	-99	-99	-99	-99	208	208	160	160	-99	-99	0.015	1	MATCH
MT-235	71	71	76	90	-99	-99	-99	-99	208	208	160	160	-99	-99	---	0.93	MATCH

Unique genotype compared against 148 samples, returning those with score>=0.857
Psib is calculated for samples that have no mismatches. Differences among samples are due to loci with missing data.

Unique genotype (63 of 109)

	MSUT.2a	MSUT.2b	G10Ja	G10Jb	MSUT.8a	MSUT.8b	MSUT.4a	MSUT.4b	UT.1a	UT.1b	UT.4a	UT.4b	UT.35a	UT.35b	Psib	Score	Type
MT-39	71	79	84	90	118	122	-99	-99	176	200	168	168	202	210			UNIQUE
MT-39	71	79	84	90	118	122	-99	-99	176	200	168	168	202	210	0.0022	1	MATCH
MT-236	71	79	84	90	118	122	-99	-99	176	200	168	168	-99	-99	0.0038	0.93	MATCH

Unique genotype compared against 148 samples, returning those with score>=0.857
Psib is calculated for samples that have no mismatches. Differences among samples are due to loci with missing data.

Unique genotype (64 of 109)

	MSUT.2a	MSUT.2b	G10Ja	G10Jb	MSUT.8a	MSUT.8b	MSUT.4a	MSUT.4b	UT.1a	UT.1b	UT.4a	UT.4b	UT.35a	UT.35b	Psib	Score	Type
MT-41	71	85	82	92	112	128	93	95	176	188	152	164	206	214			UNIQUE
MT-41	71	85	82	92	112	128	93	95	176	188	152	164	206	214	0.00051	1	MATCH

Unique genotype compared against 148 samples, returning those with score>=0.857
Psib is calculated for samples that have no mismatches. Differences among samples are due to loci with missing data.

Unique genotype (65 of 109)

	MSUT.2a	MSUT.2b	G10Ja	G10Jb	MSUT.8a	MSUT.8b	MSUT.4a	MSUT.4b	UT.1a	UT.1b	UT.4a	UT.4b	UT.35a	UT.35b	Psib	Score	Type
MT-43	71	81	90	94	-99	-99	93	95	-99	-99	160	160	202	210			UNIQUE
MT-43	71	81	90	94	-99	-99	93	95	-99	-99	160	160	202	210	0.0091	1	MATCH

Unique genotype compared against 148 samples, returning those with score>=0.857
Psib is calculated for samples that have no mismatches. Differences among samples are due to loci with missing data.

Unique genotype (66 of 109)

	MSUT.2a	MSUT.2b	G10Ja	G10Jb	MSUT.8a	MSUT.8b	MSUT.4a	MSUT.4b	UT.1a	UT.1b	UT.4a	UT.4b	UT.35a	UT.35b	Psib	Score	Type
MT-44	71	81	82	82	-99	-99	83	85	-99	-99	152	160	202	210			UNIQUE
MT-44	71	81	82	82	-99	-99	83	85	-99	-99	152	160	202	210	0.0074	1	MATCH

MT-237	71	81	82	82	118	118	83	85	-99	-99	152	160	202	210	0.0074	0.93	MATCH

Unique genotype compared against 148 samples, returning those with score>=0.857
Psib is calculated for samples that have no mismatches. Differences among samples are due to loci with missing data.

Unique genotype (67 of 109)

	MSUT.2a	MSUT.2b	G10Ja	G10Jb	MSUT.8a	MSUT.8b	MSUT.4a	MSUT.4b	UT.1a	UT.1b	UT.4a	UT.4b	UT.35a	UT.35b	Psib	Score	Type
MT-46	71	81	78	90	-99	-99	87	93	-99	-99	-99	-99	202	210			UNIQUE
MT-46	71	81	78	90	-99	-99	87	93	-99	-99	-99	-99	202	210	0.023	1	MATCH
MT-261	71	81	78	90	-99	-99	87	93	-99	-99	-99	-99	202	210	0.023	1	MATCH
MT-238	71	81	78	90	-99	-99	87	93	172	176	-99	-99	202	210	0.023	0.93	MATCH

Unique genotype compared against 148 samples, returning those with score>=0.857
Psib is calculated for samples that have no mismatches. Differences among samples are due to loci with missing data.

Unique genotype (68 of 109)

	MSUT.2a	MSUT.2b	G10Ja	G10Jb	MSUT.8a	MSUT.8b	MSUT.4a	MSUT.4b	UT.1a	UT.1b	UT.4a	UT.4b	UT.35a	UT.35b	Psib	Score	Type
MT-47	71	71	90	90	-99	-99	93	93	-99	-99	160	168	202	210			UNIQUE
MT-47	71	71	90	90	-99	-99	93	93	-99	-99	160	168	202	210	0.013	1	MATCH

Unique genotype compared against 148 samples, returning those with score>=0.857
Psib is calculated for samples that have no mismatches. Differences among samples are due to loci with missing data.

Unique genotype (69 of 109)

	MSUT.2a	MSUT.2b	G10Ja	G10Jb	MSUT.8a	MSUT.8b	MSUT.4a	MSUT.4b	UT.1a	UT.1b	UT.4a	UT.4b	UT.35a	UT.35b	Psib	Score	Type
MT-48	71	71	76	92	-99	-99	91	93	168	208	164	164	202	210			UNIQUE
MT-48	71	71	76	92	-99	-99	91	93	168	208	164	164	202	210	0.0027	1	MATCH

Unique genotype compared against 148 samples, returning those with score>=0.857
Psib is calculated for samples that have no mismatches. Differences among samples are due to loci with missing data.

Unique genotype (70 of 109)

	MSUT.2a	MSUT.2b	G10Ja	G10Jb	MSUT.8a	MSUT.8b	MSUT.4a	MSUT.4b	UT.1a	UT.1b	UT.4a	UT.4b	UT.35a	UT.35b	Psib	Score	Type
MT-49	71	87	96	96	108	128	87	95	168	176	164	172	202	210			UNIQUE
MT-49	71	87	96	96	108	128	87	95	168	176	164	172	202	210	0.00064	1	MATCH

Unique genotype compared against 148 samples, returning those with score>=0.857
Psib is calculated for samples that have no mismatches. Differences among samples are due to loci with missing data.

Unique genotype (71 of 109)

	MSUT.2a	MSUT.2b	G10Ja	G10Jb	MSUT.8a	MSUT.8b	MSUT.4a	MSUT.4b	UT.1a	UT.1b	UT.4a	UT.4b	UT.35a	UT.35b	Psib	Score	Type
MT-5	71	79	92	94	122	122	83	85	-99	-99	156	168	-99	-99			UNIQUE
MT-5	71	79	92	94	122	122	83	85	-99	-99	156	168	-99	-99	0.0025	1	MATCH
MT-229	-99	-99	92	94	122	122	83	85	-99	-99	156	168	-99	-99	0.0061	0.93	MATCH

Unique genotype compared against 148 samples, returning those with score>=0.857
Psib is calculated for samples that have no mismatches. Differences among samples are due to loci with missing data.

Unique genotype (72 of 109)

	MSUT.2a	MSUT.2b	G10Ja	G10Jb	MSUT.8a	MSUT.8b	MSUT.4a	MSUT.4b	UT.1a	UT.1b	UT.4a	UT.4b	UT.35a	UT.35b	Psib	Score	Type
MT-50	71	71	88	88	108	108	87	109	172	172	-99	-99	202	210			UNIQUE
MT-50	71	71	88	88	108	108	87	109	172	172	-99	-99	202	210	0.0028	1	MATCH
MT-262	71	71	88	88	108	108	87	109	172	172	-99	-99	202	210	0.0028	1	MATCH
MT-239	71	71	88	88	108	108	87	109	172	172	-99	-99	210	210	---	0.93	MATCH

Unique genotype compared against 148 samples, returning those with score>=0.857
Psib is calculated for samples that have no mismatches. Differences among samples are due to loci with missing data.

Unique genotype (73 of 109)

	MSUT.2a	MSUT.2b	G10Ja	G10Jb	MSUT.8a	MSUT.8b	MSUT.4a	MSUT.4b	UT.1a	UT.1b	UT.4a	UT.4b	UT.35a	UT.35b	Psib	Score	Type
MT-51	71	71	92	92	100	108	-99	-99	172	172	152	160	202	210			UNIQUE
MT-51	71	71	92	92	100	108	-99	-99	172	172	152	160	202	210	0.004	1	MATCH
MT-240	71	71	92	92	100	108	-99	-99	172	172	152	160	202	210	0.004	1	MATCH

Unique genotype compared against 148 samples, returning those with score>=0.857
Psib is calculated for samples that have no mismatches. Differences among samples are due to loci with missing data.

Unique genotype (74 of 109)

	MSUT.2a	MSUT.2b	G10Ja	G10Jb	MSUT.8a	MSUT.8b	MSUT.4a	MSUT.4b	UT.1a	UT.1b	UT.4a	UT.4b	UT.35a	UT.35b	Psib	Score	Type
MT-52	71	85	80	90	108	114	-99	-99	176	176	160	164	202	210			UNIQUE
MT-52	71	85	80	90	108	114	-99	-99	176	176	160	164	202	210	0.0044	1	MATCH

Unique genotype compared against 148 samples, returning those with score>=0.857
Psib is calculated for samples that have no mismatches. Differences among samples are due to loci with missing data.

Unique genotype (75 of 109)

	MSUT.2a	MSUT.2b	G10Ja	G10Jb	MSUT.8a	MSUT.8b	MSUT.4a	MSUT.4b	UT.1a	UT.1b	UT.4a	UT.4b	UT.35a	UT.35b	Psib	Score	Type
MT-53	71	71	68	78	108	108	69	73	172	172	160	160	202	210			UNIQUE
MT-53	71	71	68	78	108	108	69	73	172	172	160	160	202	210	0.00086	1	MATCH

Unique genotype compared against 148 samples, returning those with score>=0.857
Psib is calculated for samples that have no mismatches. Differences among samples are due to loci with missing data.

Unique genotype (76 of 109)

	MSUT.2a	MSUT.2b	G10Ja	G10Jb	MSUT.8a	MSUT.8b	MSUT.4a	MSUT.4b	UT.1a	UT.1b	UT.4a	UT.4b	UT.35a	UT.35b	Psib	Score	Type
MT-54	-99	-99	-99	-99	-99	-99	91	99	176	176	-99	-99	202	210			UNIQUE
MT-54	-99	-99	-99	-99	-99	-99	91	99	176	176	-99	-99	202	210	0.081	1	MATCH
MT-241	-99	-99	-99	-99	-99	-99	91	91	176	176	-99	-99	202	210	---	0.93	MATCH

Unique genotype compared against 148 samples, returning those with score>=0.857
Psib is calculated for samples that have no mismatches. Differences among samples are due to loci with missing data.

Unique genotype (77 of 109)

	MSUT.2a	MSUT.2b	G10Ja	G10Jb	MSUT.8a	MSUT.8b	MSUT.4a	MSUT.4b	UT.1a	UT.1b	UT.4a	UT.4b	UT.35a	UT.35b	Psib	Score	Type
MT-55	71	75	76	76	108	108	87	91	176	176	152	152	202	210			UNIQUE
MT-55	71	75	76	76	108	108	87	91	176	176	152	152	202	210	0.0014	1	MATCH
MT-242	71	75	76	76	108	108	87	91	176	176	152	152	202	202	---	0.93	MATCH

Unique genotype compared against 148 samples, returning those with score>=0.857
Psib is calculated for samples that have no mismatches. Differences among samples are due to loci with missing data.

Unique genotype (78 of 109)

	MSUT.2a	MSUT.2b	G10Ja	G10Jb	MSUT.8a	MSUT.8b	MSUT.4a	MSUT.4b	UT.1a	UT.1b	UT.4a	UT.4b	UT.35a	UT.35b	Psib	Score	Type
MT-56	71	71	70	90	120	120	97	97	172	176	160	168	202	210			UNIQUE
MT-56	71	71	70	90	120	120	97	97	172	176	160	168	202	210	0.0011	1	MATCH
MT-243	71	79	70	90	120	120	97	97	172	176	160	168	202	210	---	0.93	MATCH

Unique genotype compared against 148 samples, returning those with score>=0.857
Psib is calculated for samples that have no mismatches. Differences among samples are due to loci with missing data.

Unique genotype (79 of 109)

	MSUT.2a	MSUT.2b	G10Ja	G10Jb	MSUT.8a	MSUT.8b	MSUT.4a	MSUT.4b	UT.1a	UT.1b	UT.4a	UT.4b	UT.35a	UT.35b	Psib	Score	Type
MT-57	71	79	96	98	108	108	95	95	176	176	164	168	202	210			UNIQUE
MT-57	71	79	96	98	108	108	95	95	176	176	164	168	202	210	0.00097	1	MATCH
MT-263	71	79	96	98	108	108	95	95	176	176	164	168	202	210	0.00097	1	MATCH
MT-244	71	79	96	98	108	108	95	95	-99	-99	164	168	202	210	0.0022	0.93	MATCH

Unique genotype compared against 148 samples, returning those with score>=0.857
Psib is calculated for samples that have no mismatches. Differences among samples are due to loci with missing data.

Unique genotype (80 of 109)

	MSUT.2a	MSUT.2b	G10Ja	G10Jb	MSUT.8a	MSUT.8b	MSUT.4a	MSUT.4b	UT.1a	UT.1b	UT.4a	UT.4b	UT.35a	UT.35b	Psib	Score	Type
MT-58	85	85	88	92	110	114	95	95	176	212	160	160	202	210			UNIQUE
MT-58	85	85	88	92	110	114	95	95	176	212	160	160	202	210	0.0012	1	MATCH
MT-245	-99	-99	88	92	110	114	95	95	176	212	160	160	202	210	0.003	0.93	MATCH

Unique genotype compared against 148 samples, returning those with score>=0.857
Psib is calculated for samples that have no mismatches. Differences among samples are due to loci with missing data.

Unique genotype (81 of 109)

	MSUT.2a	MSUT.2b	G10Ja	G10Jb	MSUT.8a	MSUT.8b	MSUT.4a	MSUT.4b	UT.1a	UT.1b	UT.4a	UT.4b	UT.35a	UT.35b	Psib	Score	Type
MT-59	71	85	86	92	110	118	87	93	180	208	152	164	202	210			UNIQUE
MT-59	71	85	86	92	110	118	87	93	180	208	152	164	202	210	0.0011	1	MATCH
MT-246	71	85	86	92	110	**110**	87	93	180	208	152	164	202	210	---	0.93	MATCH

Unique genotype compared against 148 samples, returning those with score>=0.857
Psib is calculated for samples that have no mismatches. Differences among samples are due to loci with missing data.

Unique genotype (82 of 109)

	MSUT.2a	MSUT.2b	G10Ja	G10Jb	MSUT.8a	MSUT.8b	MSUT.4a	MSUT.4b	UT.1a	UT.1b	UT.4a	UT.4b	UT.35a	UT.35b	Psib	Score	Type
MT-60	79	91	76	90	108	114	87	97	176	176	164	176	202	210			UNIQUE
MT-60	79	91	76	90	108	114	87	97	176	176	164	176	202	210	0.00073	1	MATCH

Unique genotype compared against 148 samples, returning those with score>=0.857
Psib is calculated for samples that have no mismatches. Differences among samples are due to loci with missing data.

Unique genotype (83 of 109)

		MSUT.2a	MSUT.2b	G10Ja	G10Jb	MSUT.8a	MSUT.8b	MSUT.4a	MSUT.4b	UT.1a	UT.1b	UT.4a	UT.4b	UT.35a	UT.35b	Psib	Score	Type
	MT-62	71	85	88	92	110	114	93	93	172	172	160	164	202	210			UNIQUE
	MT-62	71	85	88	92	110	114	93	93	172	172	160	164	202	210	0.0012	1	MATCH

Unique genotype compared against 148 samples, returning those with score>=0.857
Psib is calculated for samples that have no mismatches. Differences among samples are due to loci with missing data.

Unique genotype (84 of 109)

		MSUT.2a	MSUT.2b	G10Ja	G10Jb	MSUT.8a	MSUT.8b	MSUT.4a	MSUT.4b	UT.1a	UT.1b	UT.4a	UT.4b	UT.35a	UT.35b	Psib	Score	Type
	MT-63	71	85	90	92	108	114	87	99	208	212	148	164	-99	-99			UNIQUE
	MT-63	71	85	90	92	108	114	87	99	208	212	148	164	-99	-99	0.002	1	MATCH

Unique genotype compared against 148 samples, returning those with score>=0.857
Psib is calculated for samples that have no mismatches. Differences among samples are due to loci with missing data.

Unique genotype (85 of 109)

		MSUT.2a	MSUT.2b	G10Ja	G10Jb	MSUT.8a	MSUT.8b	MSUT.4a	MSUT.4b	UT.1a	UT.1b	UT.4a	UT.4b	UT.35a	UT.35b	Psib	Score	Type
	MT-65	85	85	86	90	108	118	91	97	172	176	152	152	-99	-99			UNIQUE
	MT-65	85	85	86	90	108	118	91	97	172	176	152	152	-99	-99	0.0023	1	MATCH

Unique genotype compared against 148 samples, returning those with score>=0.857
Psib is calculated for samples that have no mismatches. Differences among samples are due to loci with missing data.

Unique genotype (86 of 109)

		MSUT.2a	MSUT.2b	G10Ja	G10Jb	MSUT.8a	MSUT.8b	MSUT.4a	MSUT.4b	UT.1a	UT.1b	UT.4a	UT.4b	UT.35a	UT.35b	Psib	Score	Type
	MT-66	71	79	86	90	108	120	91	99	180	204	164	168	202	210			UNIQUE
	MT-66	71	79	86	90	108	120	91	99	180	204	164	168	202	210	0.00062	1	MATCH

Unique genotype compared against 148 samples, returning those with score>=0.857
Psib is calculated for samples that have no mismatches. Differences among samples are due to loci with missing data.

Unique genotype (87 of 109)

		MSUT.2a	MSUT.2b	G10Ja	G10Jb	MSUT.8a	MSUT.8b	MSUT.4a	MSUT.4b	UT.1a	UT.1b	UT.4a	UT.4b	UT.35a	UT.35b	Psib	Score	Type
	MT-67	87	87	86	92	110	112	87	97	204	208	152	160	202	210			UNIQUE
	MT-67	87	87	86	92	110	112	87	97	204	208	152	160	202	210	0.00065	1	MATCH

Unique genotype compared against 148 samples, returning those with score>=0.857
Psib is calculated for samples that have no mismatches. Differences among samples are due to loci with missing data.

Unique genotype (88 of 109)

	MSUT.2a	MSUT.2b	G10Ja	G10Jb	MSUT.8a	MSUT.8b	MSUT.4a	MSUT.4b	UT.1a	UT.1b	UT.4a	UT.4b	UT.35a	UT.35b	Psib	Score	Type
MT-68	71	87	78	92	108	118	91	97	176	208	152	160	202	210			UNIQUE
MT-68	71	87	78	92	108	118	91	97	176	208	152	160	202	210	0.0012	1	MATCH

Unique genotype compared against 148 samples, returning those with score>=0.857
Psib is calculated for samples that have no mismatches. Differences among samples are due to loci with missing data.

Unique genotype (89 of 109)

	MSUT.2a	MSUT.2b	G10Ja	G10Jb	MSUT.8a	MSUT.8b	MSUT.4a	MSUT.4b	UT.1a	UT.1b	UT.4a	UT.4b	UT.35a	UT.35b	Psib	Score	Type
MT-69	71	79	76	90	108	108	83	85	176	176	152	152	-99	-99			UNIQUE
MT-69	71	79	76	90	108	108	83	85	176	176	152	152	-99	-99	0.0024	1	MATCH

Unique genotype compared against 148 samples, returning those with score>=0.857
Psib is calculated for samples that have no mismatches. Differences among samples are due to loci with missing data.

Unique genotype (90 of 109)

	MSUT.2a	MSUT.2b	G10Ja	G10Jb	MSUT.8a	MSUT.8b	MSUT.4a	MSUT.4b	UT.1a	UT.1b	UT.4a	UT.4b	UT.35a	UT.35b	Psib	Score	Type
MT-70	85	87	84	90	108	110	91	95	176	176	160	160	202	202			UNIQUE
MT-70	85	87	84	90	108	110	91	95	176	176	160	160	202	202	0.0011	1	MATCH
MT-247	85	87	84	90	**110**	110	91	95	176	176	160	160	202	202	---	0.93	MATCH

Unique genotype compared against 148 samples, returning those with score>=0.857
Psib is calculated for samples that have no mismatches. Differences among samples are due to loci with missing data.

Unique genotype (91 of 109)

	MSUT.2a	MSUT.2b	G10Ja	G10Jb	MSUT.8a	MSUT.8b	MSUT.4a	MSUT.4b	UT.1a	UT.1b	UT.4a	UT.4b	UT.35a	UT.35b	Psib	Score	Type
MT-71	79	85	84	92	110	114	87	97	176	176	160	160	202	210			UNIQUE
MT-71	79	85	84	92	110	114	87	97	176	176	160	160	202	210	0.0012	1	MATCH
MT-248	79	85	84	92	110	114	-99	-99	176	176	160	160	202	210	0.0035	0.93	MATCH

Unique genotype compared against 148 samples, returning those with score>=0.857
Psib is calculated for samples that have no mismatches. Differences among samples are due to loci with missing data.

Unique genotype (92 of 109)

	MSUT.2a	MSUT.2b	G10Ja	G10Jb	MSUT.8a	MSUT.8b	MSUT.4a	MSUT.4b	UT.1a	UT.1b	UT.4a	UT.4b	UT.35a	UT.35b	Psib	Score	Type
MT-72	71	85	90	96	118	128	91	95	172	212	156	168	202	206			UNIQUE
MT-72	71	85	90	96	118	128	91	95	172	212	156	168	202	206	0.00059	1	MATCH

Unique genotype compared against 148 samples, returning those with score>=0.857
Psib is calculated for samples that have no mismatches. Differences among samples are due to loci with missing data.

Unique genotype (93 of 109)

	MSUT.2a	MSUT.2b	G10Ja	G10Jb	MSUT.8a	MSUT.8b	MSUT.4a	MSUT.4b	UT.1a	UT.1b	UT.4a	UT.4b	UT.35a	UT.35b	Psib	Score	Type
MT-73	85	85	76	94	110	114	91	99	208	208	152	152	-99	-99			UNIQUE
MT-73	85	85	76	94	110	114	91	99	208	208	152	152	-99	-99	0.0019	1	MATCH

Unique genotype compared against 148 samples, returning those with score>=0.857
Psib is calculated for samples that have no mismatches. Differences among samples are due to loci with missing data.

Unique genotype (94 of 109)

	MSUT.2a	MSUT.2b	G10Ja	G10Jb	MSUT.8a	MSUT.8b	MSUT.4a	MSUT.4b	UT.1a	UT.1b	UT.4a	UT.4b	UT.35a	UT.35b	Psib	Score	Type
MT-74	71	85	80	90	108	110	93	99	176	212	152	156	234	234			UNIQUE
MT-74	71	85	80	90	108	110	93	99	176	212	152	156	234	234	0.00071	1	MATCH

Unique genotype compared against 148 samples, returning those with score>=0.857
Psib is calculated for samples that have no mismatches. Differences among samples are due to loci with missing data.

Unique genotype (95 of 109)

	MSUT.2a	MSUT.2b	G10Ja	G10Jb	MSUT.8a	MSUT.8b	MSUT.4a	MSUT.4b	UT.1a	UT.1b	UT.4a	UT.4b	UT.35a	UT.35b	Psib	Score	Type
MT-75	71	79	98	98	108	110	91	95	208	212	148	152	202	210			UNIQUE
MT-75	71	79	98	98	108	110	91	95	208	212	148	152	202	210	0.0011	1	MATCH

Unique genotype compared against 148 samples, returning those with score>=0.857
Psib is calculated for samples that have no mismatches. Differences among samples are due to loci with missing data.

Unique genotype (96 of 109)

	MSUT.2a	MSUT.2b	G10Ja	G10Jb	MSUT.8a	MSUT.8b	MSUT.4a	MSUT.4b	UT.1a	UT.1b	UT.4a	UT.4b	UT.35a	UT.35b	Psib	Score	Type
MT-76	75	85	78	98	112	128	91	97	176	212	152	152	202	210			UNIQUE

MT-76	75	85	78	98	112	128	91	97	176	212	152	152	202	210	0.00089	1	MATCH
MT-249	75	85	78	98	112	128	91	97	176	176	152	152	202	210	---	0.93	MATCH

Unique genotype compared against 148 samples, returning those with score>=0.857
Psib is calculated for samples that have no mismatches. Differences among samples are due to loci with missing data.

Unique genotype (97 of 109)

	MSUT.2a	MSUT.2b	G10Ja	G10Jb	MSUT.8a	MSUT.8b	MSUT.4a	MSUT.4b	UT.1a	UT.1b	UT.4a	UT.4b	UT.35a	UT.35b	Psib	Score	Type
MT-78	71	85	90	98	110	118	93	101	208	208	152	152	202	210			UNIQUE
MT-78	71	85	90	98	110	118	93	101	208	208	152	152	202	210	0.0016	1	MATCH

Unique genotype compared against 148 samples, returning those with score>=0.857
Psib is calculated for samples that have no mismatches. Differences among samples are due to loci with missing data.

Unique genotype (98 of 109)

	MSUT.2a	MSUT.2b	G10Ja	G10Jb	MSUT.8a	MSUT.8b	MSUT.4a	MSUT.4b	UT.1a	UT.1b	UT.4a	UT.4b	UT.35a	UT.35b	Psib	Score	Type
MT-82	71	71	-99	-99	100	130	69	89	172	212	160	160	202	210			UNIQUE
MT-82	71	71	-99	-99	100	130	69	89	172	212	160	160	202	210	0.0024	1	MATCH

Unique genotype compared against 148 samples, returning those with score>=0.857
Psib is calculated for samples that have no mismatches. Differences among samples are due to loci with missing data.

Unique genotype (99 of 109)

	MSUT.2a	MSUT.2b	G10Ja	G10Jb	MSUT.8a	MSUT.8b	MSUT.4a	MSUT.4b	UT.1a	UT.1b	UT.4a	UT.4b	UT.35a	UT.35b	Psib	Score	Type
MT-84	71	85	98	98	110	114	85	91	172	172	152	152	202	202			UNIQUE
MT-84	71	85	98	98	110	114	85	91	172	172	152	152	202	202	0.0012	1	MATCH

Unique genotype compared against 148 samples, returning those with score>=0.857
Psib is calculated for samples that have no mismatches. Differences among samples are due to loci with missing data.

Unique genotype (100 of 109)

	MSUT.2a	MSUT.2b	G10Ja	G10Jb	MSUT.8a	MSUT.8b	MSUT.4a	MSUT.4b	UT.1a	UT.1b	UT.4a	UT.4b	UT.35a	UT.35b	Psib	Score	Type
MT-85	71	91	78	98	110	112	93	97	-99	-99	152	156	202	206			UNIQUE
MT-85	71	91	78	98	110	112	93	97	-99	-99	152	156	202	206	0.0019	1	MATCH

Unique genotype compared against 148 samples, returning those with score>=0.857
Psib is calculated for samples that have no mismatches. Differences among samples are due to loci with missing data.

Unique genotype (101 of 109)

	MSUT.2a	MSUT.2b	G10Ja	G10Jb	MSUT.8a	MSUT.8b	MSUT.4a	MSUT.4b	UT.1a	UT.1b	UT.4a	UT.4b	UT.35a	UT.35b	Psib	Score	Type
MT-86	71	79	98	98	110	118	93	97	176	212	152	156	202	210			UNIQUE
MT-86	71	79	98	98	110	118	93	97	176	212	152	156	202	210	0.0012	1	MATCH
MT-265	71	79	98	98	110	118	93	97	176	212	152	156	202	210	0.0012	1	MATCH
MT-251	71	79	-99	-99	110	118	93	97	176	212	152	156	202	210	0.0041	0.93	MATCH

Unique genotype compared against 148 samples, returning those with score>=0.857
Psib is calculated for samples that have no mismatches. Differences among samples are due to loci with missing data.

Unique genotype (102 of 109)

	MSUT.2a	MSUT.2b	G10Ja	G10Jb	MSUT.8a	MSUT.8b	MSUT.4a	MSUT.4b	UT.1a	UT.1b	UT.4a	UT.4b	UT.35a	UT.35b	Psib	Score	Type
MT-87	79	85	98	98	114	120	93	97	156	200	148	152	202	210			UNIQUE
MT-87	79	85	98	98	114	120	93	97	156	200	148	152	202	210	0.0007	1	MATCH
MT-252	-99	-99	98	98	114	120	93	97	156	200	148	152	202	210	0.002	0.93	MATCH

Unique genotype compared against 148 samples, returning those with score>=0.857
Psib is calculated for samples that have no mismatches. Differences among samples are due to loci with missing data.

Unique genotype (103 of 109)

	MSUT.2a	MSUT.2b	G10Ja	G10Jb	MSUT.8a	MSUT.8b	MSUT.4a	MSUT.4b	UT.1a	UT.1b	UT.4a	UT.4b	UT.35a	UT.35b	Psib	Score	Type
MT-89	71	85	98	98	110	128	97	99	184	212	148	152	202	210			UNIQUE
MT-89	71	85	98	98	110	128	97	99	184	212	148	152	202	210	0.0012	1	MATCH

Unique genotype compared against 148 samples, returning those with score>=0.857
Psib is calculated for samples that have no mismatches. Differences among samples are due to loci with missing data.

Unique genotype (104 of 109)

	MSUT.2a	MSUT.2b	G10Ja	G10Jb	MSUT.8a	MSUT.8b	MSUT.4a	MSUT.4b	UT.1a	UT.1b	UT.4a	UT.4b	UT.35a	UT.35b	Psib	Score	Type
MT-90	71	91	92	98	114	120	91	91	176	200	148	152	202	206			UNIQUE
MT-90	71	91	92	98	114	120	91	91	176	200	148	152	202	206	0.0007	1	MATCH

Unique genotype compared against 148 samples, returning those with score>=0.857
Psib is calculated for samples that have no mismatches. Differences among samples are due to loci with missing data.

Unique genotype (105 of 109)

	MSUT.2a	MSUT.2b	G10Ja	G10Jb	MSUT.8a	MSUT.8b	MSUT.4a	MSUT.4b	UT.1a	UT.1b	UT.4a	UT.4b	UT.35a	UT.35b	Psib	Score	Type
MT-91	71	79	-99	-99	112	122	87	93	176	212	-99	-99	202	210			UNIQUE
MT-91	71	79	-99	-99	112	122	87	93	176	212	-99	-99	202	210	0.0084	1	MATCH

Unique genotype compared against 148 samples, returning those with score>=0.857
Psib is calculated for samples that have no mismatches. Differences among samples are due to loci with missing data.

Unique genotype (106 of 109)

	MSUT.2a	MSUT.2b	G10Ja	G10Jb	MSUT.8a	MSUT.8b	MSUT.4a	MSUT.4b	UT.1a	UT.1b	UT.4a	UT.4b	UT.35a	UT.35b	Psib	Score	Type
MT-92	79	85	88	98	110	110	87	95	176	212	148	152	202	210			UNIQUE
MT-92	79	85	88	98	110	110	87	95	176	212	148	152	202	210	0.0012	1	MATCH
MT-253	79	85	88	98	106	110	87	95	176	212	148	152	202	210	---	0.93	MATCH

Unique genotype compared against 148 samples, returning those with score>=0.857
Psib is calculated for samples that have no mismatches. Differences among samples are due to loci with missing data.

Unique genotype (107 of 109)

	MSUT.2a	MSUT.2b	G10Ja	G10Jb	MSUT.8a	MSUT.8b	MSUT.4a	MSUT.4b	UT.1a	UT.1b	UT.4a	UT.4b	UT.35a	UT.35b	Psib	Score	Type
MT-93	71	87	-99	-99	110	118	87	95	176	212	-99	-99	202	210			UNIQUE
MT-93	71	87	-99	-99	110	118	87	95	176	212	-99	-99	202	210	0.0096	1	MATCH
MT-254	71	87	-99	-99	110	118	87	95	-99	-99	-99	-99	202	210	0.025	0.93	MATCH

Unique genotype compared against 148 samples, returning those with score>=0.857
Psib is calculated for samples that have no mismatches. Differences among samples are due to loci with missing data.

Unique genotype (108 of 109)

	MSUT.2a	MSUT.2b	G10Ja	G10Jb	MSUT.8a	MSUT.8b	MSUT.4a	MSUT.4b	UT.1a	UT.1b	UT.4a	UT.4b	UT.35a	UT.35b	Psib	Score	Type
MT-94	79	85	74	74	110	114	97	97	172	172	-99	-99	-99	-99			UNIQUE
MT-94	79	85	74	74	110	114	97	97	172	172	-99	-99	-99	-99	0.0034	1	MATCH

Unique genotype compared against 148 samples, returning those with score>=0.857
Psib is calculated for samples that have no mismatches. Differences among samples are due to loci with missing data.

Unique genotype (109 of 109)

	MSUT.2a	MSUT.2b	G10Ja	G10Jb	MSUT.8a	MSUT.8b	MSUT.4a	MSUT.4b	UT.1a	UT.1b	UT.4a	UT.4b	UT.35a	UT.35b	Psib	Score	Type

	MT-96	71	83	76	98	108	114	95	101	172	180	148	152	206	210			UNIQUE
	MT-96	71	83	76	98	108	114	95	101	172	180	148	152	206	210	0.00055	1	MATCH

Unique genotype compared against 148 samples, returning those with score>=0.857
Psib is calculated for samples that have no mismatches. Differences among samples are due to loci with missing data.

Generated by allelematch: an R package
To reference this analysis please use citation("allelematch")
