# Supplementary material for: Conflict Bear Translocation: Investigating Population Genetics and Fate of Bear Translocation in Dachigam National Park, Jammu and Kashmir, India
Source: PLoS One. 2015 Aug 12;10(8):e0132005. doi: 10.1371/journal.pone.0132005 (PMC4534036; doi:10.1371/journal.pone.0132005)
Supplement: S4 Table — (DOC) [file pone.0132005.s005.doc]

**S4 Table. Details of 11 translocated individual bears from elsewhere in the landscape to DNP**

| **Bear ID** | **Date of capture /**  **involvement in conflicts** | **Characteristics of capture site** | **Characteristics of release site** | **Date of first capture after release†** | **First genetic recapture day after translocation outside Dachigam NP** | **Bear status** | **Season** |
| --- | --- | --- | --- | --- | --- | --- | --- |
| BM1 | 27-03-2011 | Cropland | Riverine forest | 07-04-2011 | 11^th^ Day | Moved back | Spring |
| BM2 | 30-04-2011 | Orchard | Pine forest | 10-05-2011 | 10^th^ Day | Moved back | Spring |
| BM4 | 30-10-2009 | Human habitation | Mixed forest | 01-11-2009 | 15^th^ Day | Moved back | Spring |
| BM6 | 09-05-2011 | Orchard | Mixed forest | 16-05-2011 | 7^th^ Day | Moved back | Autumn |
| BM7 | 07-04-2011 | Orchard | Riverine forest | 16-04-2011 | 9^th^ Day | Moved back | Spring |
| BF9 | 08-04-2011 | Human habitation | Riverine forest | 21-04-2011 | 13^th^ Day | Moved back | Spring |
| BF10 | 20-10-2009 | Orchard | Pine forest | 28-10-2009 | 8^th^ Day | Moved back | Autumn |
| BM3 | 28-07-2011 | Human habitation | Riverine forest | - |  | Settled in Dachigam | Summer |
| BF5 | 29-10-2010 | Cropland | Mixed forest | - |  | Settled in Dachigam | Autumn |
| BF8 | 07-06-2011 | Orchard | Mixed forest | - |  | Settled in Dachigam | Summer |
| BF11 | 21-07-2009 | Human habitation | Riverine forest | - |  | Settled in Dachigam | Summer |

**†** Few dates are approximates.
