# Supplementary material for: Conflict Bear Translocation: Investigating Population Genetics and Fate of Bear Translocation in Dachigam National Park, Jammu and Kashmir, India
Source: PLoS One. 2015 Aug 12;10(8):e0132005. doi: 10.1371/journal.pone.0132005 (PMC4534036; doi:10.1371/journal.pone.0132005)
Supplement: S5 Table — (DOC) [file pone.0132005.s006.doc]

**S5 Table. Area of activity of bears which moved backward and those which get settled in DNP after translocation based on 50% kernel isopleths**

| **Individual ID** | **Type-I (area in sq. km)** | **Individual ID** | **Type-II (area in sq. km)** |
| --- | --- | --- | --- |
| BM1 | 23.2 | BM3 | 16.7 |
| BM2 | 17.7 | BF5 | 10.4 |
| BM4 | 52.3 | BF8 | 3.8 |
| BM6 | 4.1 | BF11 | 8.1 |
| BM7 | 10.5 |  |  |
| BF9 | 20.2 |  |  |
| BF10 | 8.1 |  |  |

Here, Type –I denotes those bears that moved backward to their capture sites and Type-II denotes those bears that get settled in Dachigam National Park after translocation.
