# Supplementary material for: Conflict Bear Translocation: Investigating Population Genetics and Fate of Bear Translocation in Dachigam National Park, Jammu and Kashmir, India
Source: PLoS One. 2015 Aug 12;10(8):e0132005. doi: 10.1371/journal.pone.0132005 (PMC4534036; doi:10.1371/journal.pone.0132005)
Supplement: S6 Table — (DOC) [file pone.0132005.s007.doc]

S6 Table. - Matrix of land use/land cover use rankings for Asiatic black bear based on comparing proportion of use within 95% isopleths with proportions of total available land use/ land cover types in the area of analysis in Dachigam landscape during.

+ Preference, - avoidance

Each mean element in the matrix was replaced by its sign and triple sign represents significant deviation from random at *P* < 0.5.

(MF= Mixed forest, GS= Grassland and Scrubland, PF=Pine forest, HH= Human habitation, C=Orchards/Cropland).

| **Matrix of land use/ land cover use rankings of type-I individuals** | | | | | | |
| --- | --- | --- | --- | --- | --- | --- |
| **Habitat type** | **OC** | **HH** | **PF** | **MF** | **GS** | **Rank** |
| Orchards/ croplands | 0 | +++ | +++ | +++ | +++ | 4 |
| Habitation | --- | 0 | +++ | +++ | + | 3 |
| Pine forest | --- | --- | 0 | +++ | + | 2 |
| Mixed forest | --- | - | - | 0 | +++ | 1 |
| Grassland and scrubland | --- | --- | --- | --- | 0 | 0 |

| **Matrix of land use/ land cover use rankings of type-II individuals** | | | | | | |
| --- | --- | --- | --- | --- | --- | --- |
| **Habitat type** | **MF** | **PF** | **GS** | **OC** | **HH** | **Rank** |
| Mixed forest | 0 | +++ | +++ | +++ | + | 4 |
| Pine forest | --- | 0 | - | +++ | + | 2 |
| Grassland and scrubland | --- | + | 0 | +++ | + | 3 |
| Orchards/ cropland | - | - | - | 0 | + | 1 |
| Human habitation | --- | --- | --- | --- | 0 | 0 |

Here, Type –I denotes those bears that moved backward to their first physical site of capture and Type-II denotes those bears that get settled in Dachigam National Park after translocation.
